# Anion Induced Electric Double Layer Compression and Desolvation Optimization Enable Long Life Zinc Anodes under High‐Rate

**DOI:** 10.1002/advs.202513291

**Published:** 2025-09-23

**Authors:** Xiangyu Ren, Sibo Zhao, Fang Song, Shenghong Ju, FuMing Wang, Xiaowei Yang, Yunwen Wu

**Affiliations:** ^1^ School of Material Science and Engineering Shanghai Jiao Tong University Shanghai 200240 P. R. China; ^2^ Graduate Institute of Applied Science and Technology National Taiwan University of Science and Technology Taipei 106 Taiwan; ^3^ School of Chemistry and Chemical Engineering Shanghai Jiao Tong University Shanghai 200240 P. R. China

**Keywords:** aqueous zinc‐ion batteries, electric double layer, high rate, ionic additive, long life

## Abstract

Aqueous zinc‐ion batteries (AZIBs) represent a promising next‐generation energy storage solution. However, AZIBs suffer from severe dendrite growth caused by rampant Zn^2^⁺ 2D diffusion and sluggish desolvation kinetics, thus exhibiting extremely short cycle life under high‐rate conditions. Here in, a novel additive DL‐O‐Methylserine (MeSer) is reported, which effectively optimizes Zn^2^⁺ diffusion behavior and facilitates the desolvation process. Experimental and computational results reveal that MeSer^−^ adsorption on the electrode surface compresses the electric double layer (EDL), thereby reducing repulsive forces within it. The decrease in repulsion further enhances Zn^2^⁺ 3D diffusion leading to uniform deposition. Furthermore, MeSer^−^ interacts with Zn^2^⁺ located in solvation sheath, reducing desolvation energy barriers and improving rate capability. Consequently, Zn||Zn symmetric cells with MeSer exhibits superior cycling stability of 2320 h under 5 mA cm^−2^ and 5 mA h cm^−2^ and can endure extreme high‐current conditions (20 mA cm^−2^, 20 mA h cm^−2^) for up to 600 h, such performance exceeds most of the previously documented results. The Zn||V_2_O_5_ full cells maintained 86% capacity retention after 3500 cycles at 5 A g^−1^. This work demonstrates the remarkable effectiveness of a simple EDL regulation strategy in enhancing AZIB performance.

## Graphical abstract

Here, the ionic additive MeSer compresses the EDL, reducing its thickness and internal repulsion, thereby enhancing 3D diffusion to achieve smooth Zn deposition. Meanwhile, MeSer^−^ promote Zn^2^⁺ desolvation within the EDL, improving rate performance. Consequently, the symmetric cell operates for 600 h at 20 mA cm^−2^ and 20 mA h cm^−2^, and the full cell stably cycles over 3500 cycles at 5 A g^−1^.

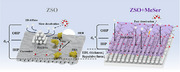

## 1 Introduction

Amid worsening global environmental pollution and an urgent demand for integrating renewable energy, large‐scale energy storage systems continue to experience rapid growth.^[^ ^1^, ^2^ ^]^ Aqueous battery systems are highly promising due to their low cost and inherent non‐flammability.^[^ ^3^, ^4^, ^5^ ^]^ Lead‐acid batteries currently dominate these systems, yet their low energy density and considerable toxicity limit their application.^[^ ^6^ ^]^ Aqueous zinc‐ion batteries (AZIBs) have emerged as promising alternatives, offering a high theoretical specific capacity (820 mA h g^−1^ or 5851 mA h mL^−1^), abundant zinc resources, environmental sustainability, and a favorable redox potential (0.76 V vs SHE).^[^ ^7^, ^8^, ^9^, ^10^ ^]^

In solution, Zn^2+^ associate with six H_2_O molecules ([Zn(H_2_O)_6_]^2^⁺) and undergo continuous dissolution/desolvation throughout charge‐discharge cycling.^[^ ^11^, ^12^ ^]^ During deposition, desolvated H_2_O molecule adhere to the anode surface, triggering parasitic reactions such as hydrogen evolution reaction (HER) and Zn_4_(OH)_6_SO_4_·xH_2_O (ZHS).^[^ ^13^ ^]^ And slow dissolution/desolvation negatively affects the rate performance of the AZIBs.^[^ ^14^ ^]^ Moreover, spontaneous 2D diffusion of Zn^2+^ toward protruding sites during deposition promotes uncontrolled dendrite growth.^[^ ^15^ ^]^ Solving these issues is essential for the successful commercialization of AZIBs.

As zinc enters the electrolyte, an electric double layer (EDL) develops at the electrode/electrolyte interface, serving as the site for Zn^2^⁺ desolvation and deposition.^[^ ^16^ ^]^ The EDL structure plays a decisive role in electrochemical reactions at the interface. According to the Derjaguin–Landau–Verwey–Overbeek (DLVO) theory, Zn^2^⁺ in aqueous solution is influenced by both electrostatic repulsion from ions within the EDL and Van Der Waals (VDW) attraction.^[^ ^17^, ^18^ ^]^ Under repulsive forces, Zn^2^⁺ undergoes 2D diffusion toward protruding tips. While attractive forces facilitate 3D diffusion, resulting in a dense deposition layer. According to the Poisson–Boltzmann equation, a thinner EDL reflects weaker electrostatic repulsion.^[^ ^18^, ^19^ ^]^ Therefore, decreasing EDL thickness to weaken repulsion while enhancing ionic attraction may optimize Zn^2^⁺ deposition kinetics.

The regulation of the EDL is primarily conducted through the design of eutectic electrolytes,^[^ ^20^ ^]^ artificial solid electrolyte interfaces (SEI),^[^ ^14^ ^]^ and the introduction of additives.^[^ ^21^ ^]^ Introducing additives is an effective, economical, and rapid strategy for improving the rate capability and stability of AZIBs.^[^ ^22^ ^]^ Weng incorporated [EMIM]OTF into the EDL to expel H_2_O molecules at the interface,^[^ ^21^ ^]^ promoting long‐term stable cycling of the zinc anode—this is currently the mainstream perspective on the role of additives in EDL modulation. Huang studied 15 different additives as EDL regulators,^[^ ^23^ ^]^ systematically investigating their molecular physical properties and effects on battery performance. Existing studies generally agree that EDL modulation helps suppress side reactions,^[^ ^24^, ^25^ ^]^ but there is a lack of in‐depth exploration of its specific effects on metal ion diffusion and desolvation processes. Consequently, there is an urgent need to develop an additive molecule specifically designed to act on the EDL, enabling further exploration of EDL critical role in battery performance and functional mechanisms.

**Figure 1:**
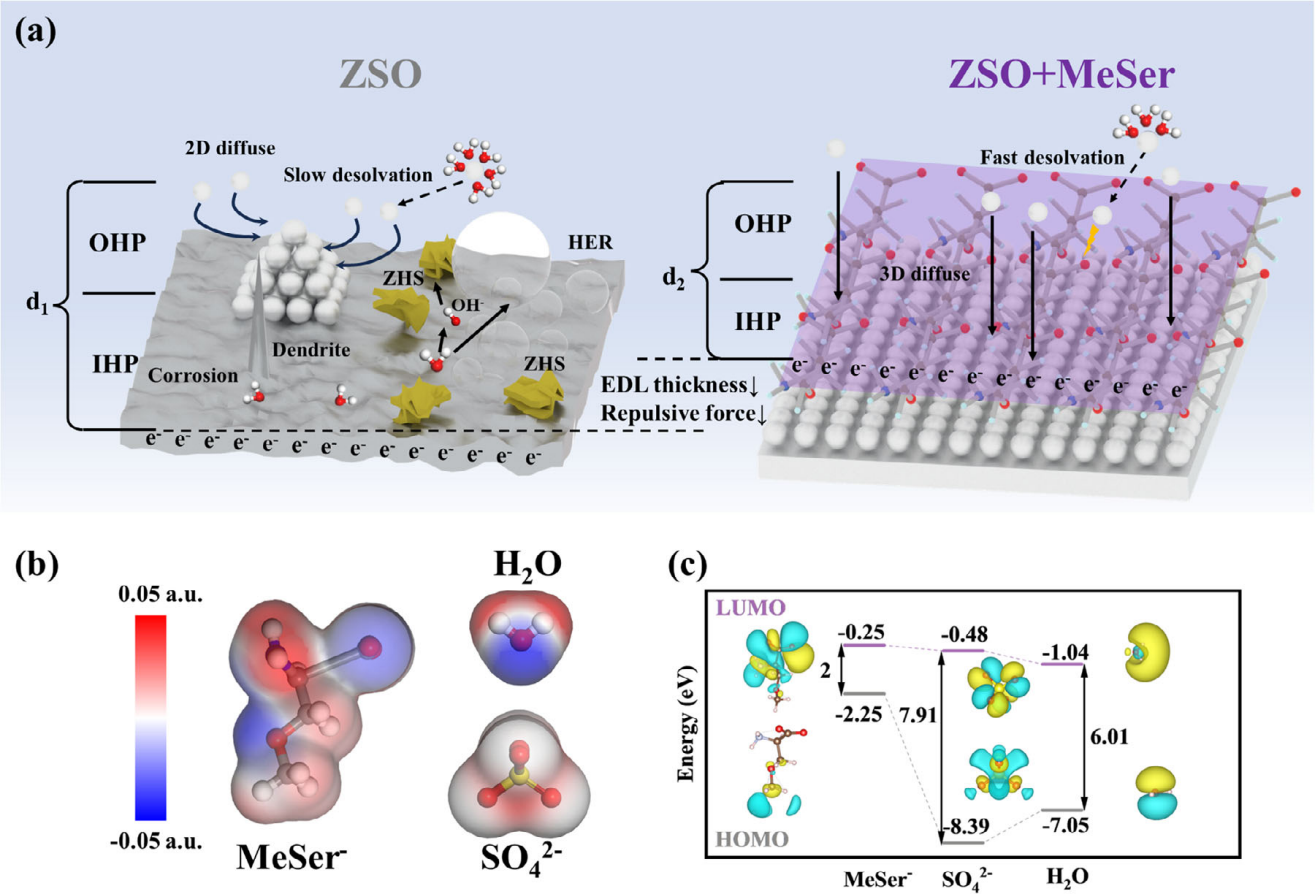
a) Schematic illustration of MeSer^−^ regulation on EDL and Zn^2^⁺ desolvation process. b) Molecular surface electrostatic potential of MeSer^−^, SO_4_^2,−^, and H_2_O. c) Molecular orbital energies of MeSer^−^, SO_4_^2−^, and H_2_O.

Here, we report a novel additive, DL‐O‐Methylserine (MeSer), which effectively reduces EDL thickness, suppresses repulsive interactions and improves Zn^2^⁺ 3D diffusion (**Figure** **1**). Extensive experiments and first‐principles calculations elucidate its mechanisms at the electrode/electrolyte interface. In solution, MeSer spontaneously dissociates into MeSer^−^ and proton (H⁺), MeSer^−^ compresses the EDL by altering the interfacial potential, thereby reducing repulsive forces within the EDL and enhancing Zn^2^⁺ 3D diffusion. The electrostatic interaction of MeSer^−^ with Zn^2^⁺ in the solvation sheath accelerated the desolvation of Zn^2^⁺, significantly enhancing battery rate performance. As a result, MeSer enabled symmetric cells to sustain over 2320 h of stable cycling at 5 mA cm^−2^ and 5 mA h cm^−2^. Even under severe conditions of 20 mA cm^−2^ and 20 mA h cm^−2^, the cycle life extends to 600 h—exceeding most prior studies. The Zn||V_2_O_5_ full cell assembled with this strategy successfully cycles for over 3500 cycles at 5 A g^−1^ with a capacity retention of 86%. This work offers fresh perspectives on EDL additive mechanisms and introduces a promising new additive.

## 2 Results and Discussion

To begin, we investigated the fundamental properties of MeSer^−^, H_2_O and SO_4_^2^^−^, as well as the changes in the solvation structure of the electrolyte before and after additive introduction. The solution pH decreased from 4.3 to 3.5 after configuring 2M ZnSO_4_ (ZSO) into ZSO+150mM MeSer (ZSO+MeSer), indicating that MeSer ionized in solution and producing MeSer^−^ and H⁺ (Figure S1, Supporting Information). Simulation results show that MeSer^−^ possesses multiple sites capable of interacting with Zn^2^⁺, indicating a stronger tendency for adsorption on the zinc anode (Figure 1b).^[^ ^26^ ^]^ Molecular orbital energies show that MeSer^−^ has the smallest energy gap (2 eV) than SO_4_^2^^−^ (7.91 eV) and H_2_O (6.01 eV), which meant that MeSer^−^ has the strongest electron transfer ability (Figure 1c).^[^ ^27^ ^]^ These computational results indicated that MeSer^−^ has the greatest potential as additive for AZIB. MeSer^−^ may enter the solvation sheath of Zn^2^⁺ through electrostatic interactions. Molecular dynamics (MD) simulations were conducted to analyze the solvation structures of the electrolyte system before and after MeSer introduction. For ZSO electrolytes, the radial distribution function (RDF) and coordination number (CN) analysis suggest that SO_4_^2^^−^ and H_2_O molecules enter the first solvation shell and coordinate with Zn^2^⁺. SO_4_^2^^−^ exhibits a CN of 0.77, whereas H_2_O molecules have a CN of 5.25, implying that the first solvation sheath comprises approximately five H_2_O molecules and one SO_4_^2^^−^. Following the introduction of MeSer, the probability of MeSer^−^ appearing at a distance of 1.8 Å from Zn^2^⁺ increases. However, given its low concentration, MeSer^−^ hardly coordinates with Zn^2^⁺, with a CN of merely 0.2. This suggests that MeSer^−^ has little impact on Zn^2^⁺ solvation structure.

**Figure 2:**
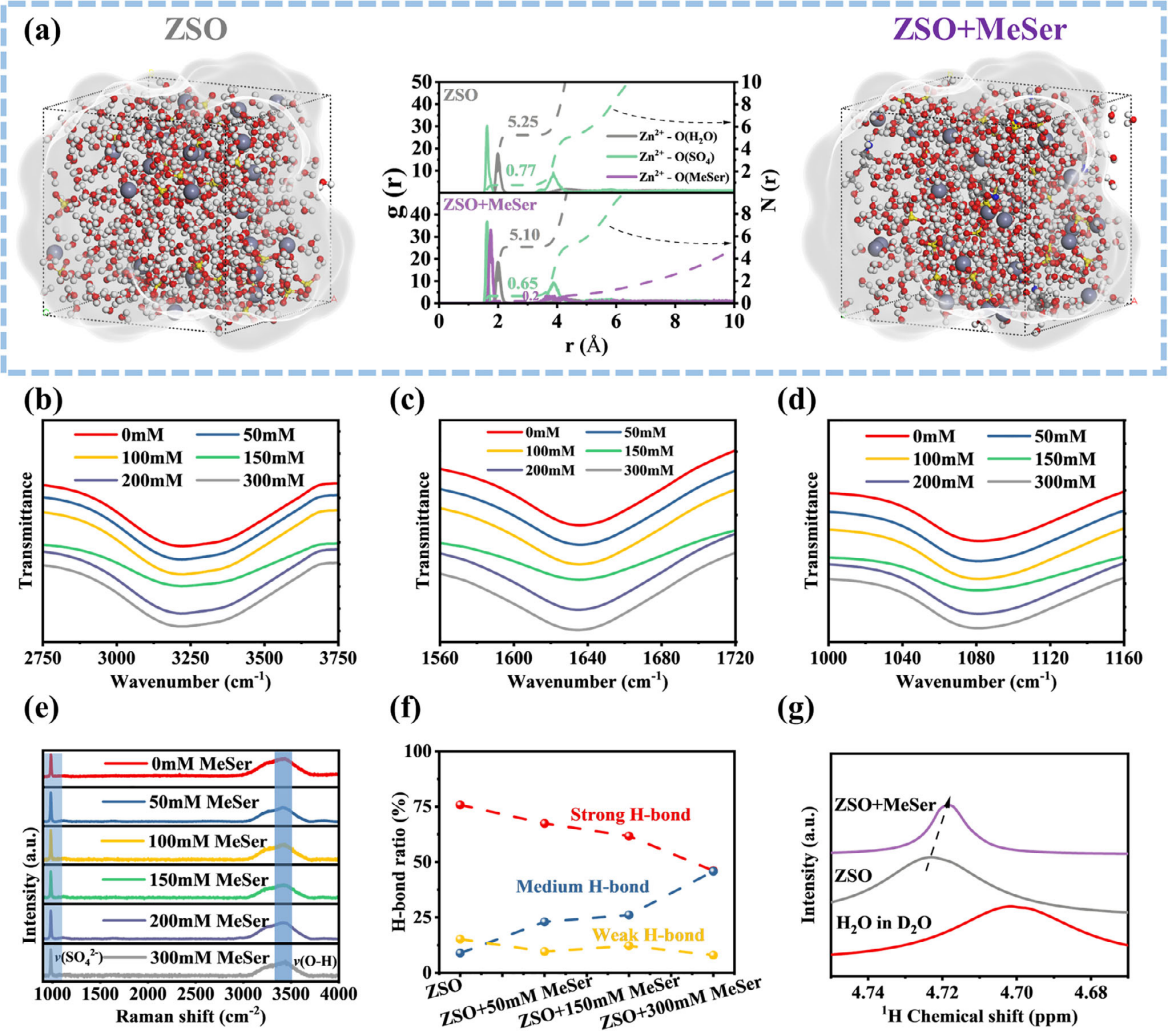
a) Snapshots, RDF and CN analysis of the MD simulations of ZSO and ZSO+MeSer electrolyte. b–d) FTIR spectra of ZSO electrolytes with different MeSer concentrations. e) Raman spectra of ZSO electrolytes with different MeSer concentrations. f) Summary of H‐bond in different electrolytes. g) 1H NMR spectra of H_2_O and different electrolytes.

To further clarify the role of MeSer in Zn^2^⁺ solvation, Fourier transform infrared (FTIR) spectroscopy and Raman spectra were conducted. The changes in Zn^2^⁺ solvation structure can be analyzed through the characterization of O‐H stretching vibrations and SO_4_^2^^−^ vibrations.^[^ ^28^ ^]^ **Figure** **2**b–d present the O‐H stretching vibration, O‐H bending vibration, and SO_4_^2−^ vibration, revealing that MeSer introduction does not induce significant peak shifts. Similarly, Raman spectroscopy results indicate that the vibrational peaks of SO_4_^2^^−^ and O‐H remain largely unchanged (Figure 2e). These observations demonstrate that 300mM MeSer does not substantially modify the solvation structure, allowing for a more accurate evaluation of interfacial electrochemistry's effects on zinc anode performance. The O‐H stretching vibration peak in the Raman spectrum can be decomposed into three separate peaks to provide a detailed explanation of spectral (Figure S2, Supporting Information). The peak at approximately 3209.6 cm^−1^ corresponds to strong H‐bonds within the H_2_O molecular framework. The peak near 3394.9 cm^−1^ represents intermediate H‐bonds among H_2_O molecules with limited connectivity. The peak at 3557.8 cm^−1^ is attributed to weak H─bonds within polymeric H_2_O structures, where molecular connections are relatively loose.^[^ ^29^ ^]^ Figure 2f illustrates the variation in H‐bond distribution, showing that increasing the additive concentration reduces the proportion of strong H‐bonds from 75.8% to 46.1%, while moderate H‐bonds increase from 8.9% to 45.8%, demonstrating the additive‐induced restructuring of the H_2_O H‐bond network. Nuclear magnetic resonance (NMR) results reinforce this conclusion. The ^1^H peak shifts downfield upon incorporating ZnSO_4_ into H_2_O, indicating a decrease in the electron cloud density around the H, leading to enhanced deshielding effects.^[^ ^30^ ^]^ The ^1^H peak moves upfield after MeSer incorporation, reflecting an increase in electron density that strengthens the shielding effect—potentially driven by interactions between MeSer^−^ and H atoms. Clearly, MeSer does not significantly affect the solvation structure of the solution. Therefore, it is necessary to explore other key factors that may influence battery performance.

**Figure 3:**
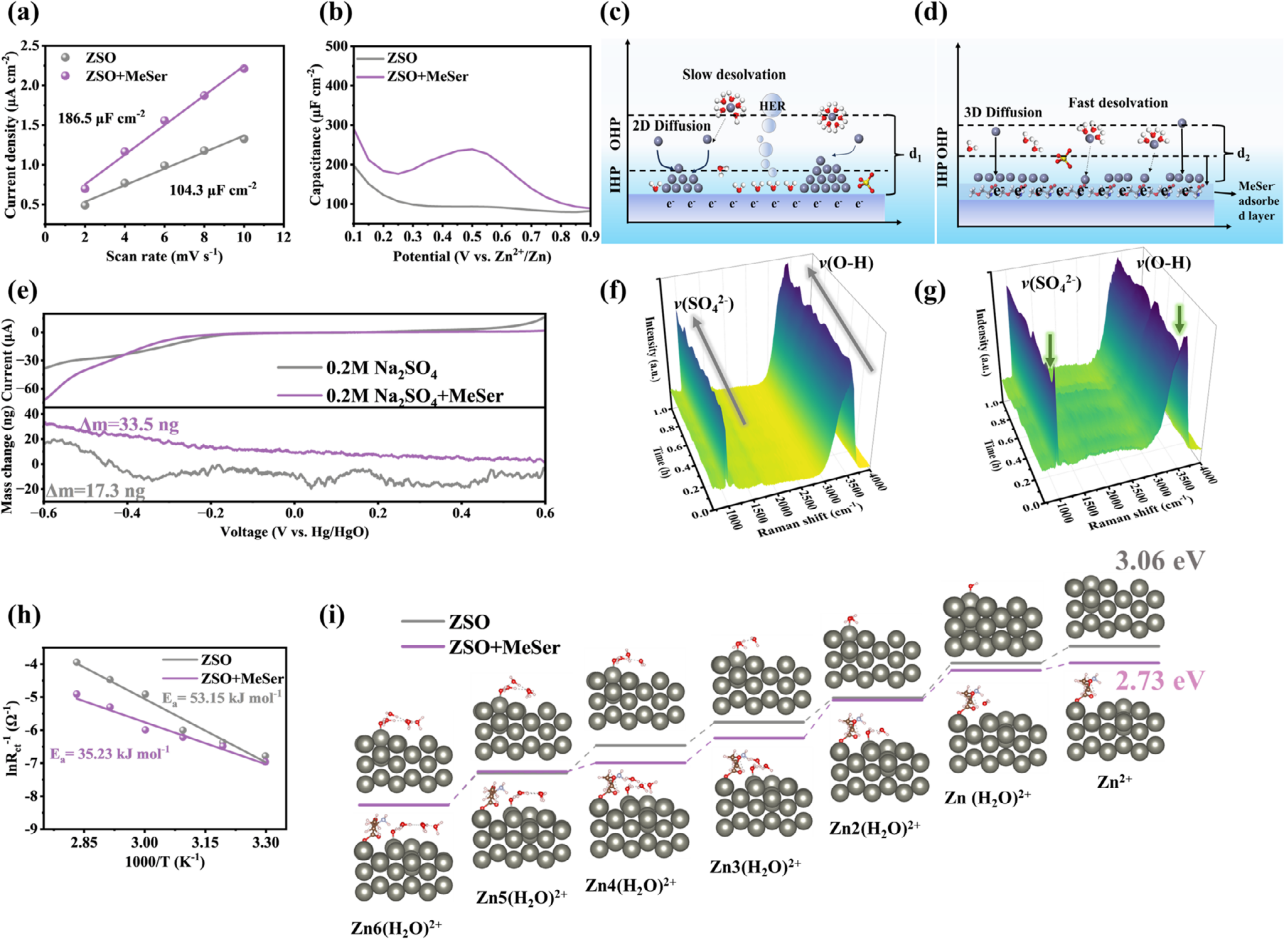
a) EDLC tests. b) DC tests. Schematic illustration of the electrode interface EDL, Zn^2^⁺ deposition process, and desolvation process c)before and d)after incorporating MeSer. e) EQCM‐measured mass change of the electrode during scanning from 0.6 V to ‐0.6 V. In situ Raman spectra of zinc anode during cycling in f)ZSO and g)ZSO+MeSer. h) desolvation activation energy tests of the zinc anode in different electrolytes. i) DFT‐calculated desolvation energy barrier of Zn(6H_2_O)^2^⁺ on bare zinc and zinc with adsorbed MeSer^−^.

Given that MeSer does not induce major changes in electrolyte properties, we proceeded to examine its impact on the electrode/electrolyte interface. Zn^2^⁺ desolvation and deposition take place within the EDL, necessitating an in‐depth analysis of its structure to understand Zn^2^⁺ interfacial dynamics. Electric double layer capacitance (EDLC) and differential capacitance (DC) were utilized to quantify EDL modifications due to MeSer introduction. Through fitting cyclic voltammetry (CV) data from multiple scan rates, we obtained EDLC values (**Figure** **3**a; Figure S3, Supporting Information). The EDLC of the zinc anode in ZSO measured 104.3 µF cm^−2^ but increased significantly to 186.5 µF cm^−2^ after MeSer addition. Similarly, the DC curve in Figure 3b exhibits a matching trend, where ZSO+MeSer displays higher DC values compared to ZSO over the 0.1–0.9 V range. Based on the equation C = εA/d, where ε is the dielectric constant of the electrolyte, A is the electrode surface area, and d represents EDL thickness, the increase in C implies a decrease in d.^[^ ^18^ ^]^ The decrease in d signifies a reduction in repulsive forces within the EDL, which in turn facilitates the occurrence of Zn^2^⁺ 3D diffusion. Figure 3c, d illustrate the effects of MeSer^−^ on EDL compression (d_2_<d_1_), Zn^2^⁺ deposition kinetics and desolvation behavior. Density functional theory (DFT) simulations were further conducted to confirm the adsorption state of MeSer^−^ on the electrode surface. The most favorable adsorption geometry of MeSer^−^ was first computed, followed by adsorption energy evaluations on (002), (100), and (101) crystal planes (Figures S4 and S5, Supporting Information). Findings suggest MeSer^−^ preferentially binds to the (100) plane (‐4.29 eV > ‐3.05 eV > ‐2.75 eV). Additional calculations show that MeSer^−^ exhibits stronger affinity for the (100) surface compared to H_2_O molecules (Figure S5, Supporting Information). This indicates that MeSer^−^ readily assembles at the electrode interface, integrating into the EDL and displacing surface H_2_O molecules, beneficial for side reaction suppression.^[^ ^31^ ^]^

We further utilized advanced experimental techniques to verify whether MeSer adsorbs onto the electrode surface. The in situ electrochemical quartz crystal microbalance (EQCM) measurements based on linear sweep voltammetry (LSV) further confirm that MeSer^−^ can adsorb onto the surface (Figure 3e). To avoid mass changes related to zinc deposition, 0.2M Na_2_SO_4_ was used as the base electrolyte. The Au electrode was scanned from 0.6 V to ‐0.6 V at a rate of 10 mV s^−1^ in both Na_2_SO_4_ and Na_2_SO_4_+MeSer solutions. In Na_2_SO_4_, the Au electrode mass fluctuated significantly as voltage decreased, with only a minor increase of 17.3 ng at ‐0.6 V, suggesting the absence of adsorption. In Na_2_SO_4_+MeSer, the Au electrode mass continuously rose, reaching 33.5 ng at ‐0.6 V, indicating stable MeSer^−^ adsorption.^[^ ^32^ ^]^ Furthermore, in situ Raman spectroscopy was employed to investigate interfacial changes during plating/stripping at 1 mA cm^−2^. In ZSO electrolyte, the characteristic peaks of SO_4_^2^^−^ and H_2_O continuously intensified over time, indicating the sustained accumulation of SO_4_^2^^−^ and H_2_O at the interface (Figure 3f; Figure S6, Supporting Information).^[^ ^33^ ^]^ Within the ZSO+MeSer electrolyte, the characteristic peaks of SO_4_^2^^−^ and H_2_O exhibited a sharp decline at the onset of plating, signifying their expulsion from the inner EDL region (Figure 3g; Figure S6, Supporting Information). Thanks to MeSer^−^ displacing SO_4_^2^^−^ and H_2_O from the inner EDL, the characteristic peaks intensity of SO_4_^2^^−^ and H_2_O not significantly increased compared to the initial state. These in situ experimental findings offer strong validation of MeSer^−^ adsorption at the electrode interface, substantiating its contribution to EDL compression.

As EDL is the key region where desolvation takes place, its structural modifications inevitably influence the desolvation process. We measured the desolvation activation energy of different electrolytes, collecting Nyquist plots within the 30 °C–80 °C range for fitting analysis (Figure S7, Supporting Information). The fitting results in Figure 3h show that the desolvation activation energy for ZSO electrolyte is 53.15 kJ mol^−1^, which significantly decreases to 35.23 kJ mol^−1^ upon MeSer addition. This reduction in activation energy facilitates enhanced reaction kinetics, improving battery rate performance. MeSer^−^ within the EDL facilitates Zn^2^⁺ desolvation from its solvation shell via electrostatic interactions. To further investigate anionic adsorption effects on desolvation thermodynamics, we conducted precise DFT simulations. A Zn(H_2_O)_6_^2^⁺ cluster was placed on both bare zinc and zinc adsorbed with MeSer^−^, and the energy barriers for each step in Zn(H_2_O)_6_^2^⁺ desolvation to Zn^2^⁺ were calculated. The DFT results in Figure 3i indicate that the total desolvation energy barrier for Zn(H_2_O)_6_^2^⁺ on bare zinc is 3.06 eV, which decreases to 2.73 eV after MeSer^−^ adsorption. These DFT calculations strongly support experimental results and clearly demonstrate that MeSer^−^ within the EDL enhance Zn^2^⁺ desolvation. The combined experimental and computational results robustly confirm that MeSer^−^ within EDL facilitates Zn^2^⁺ desolvation.

**Figure 4:**
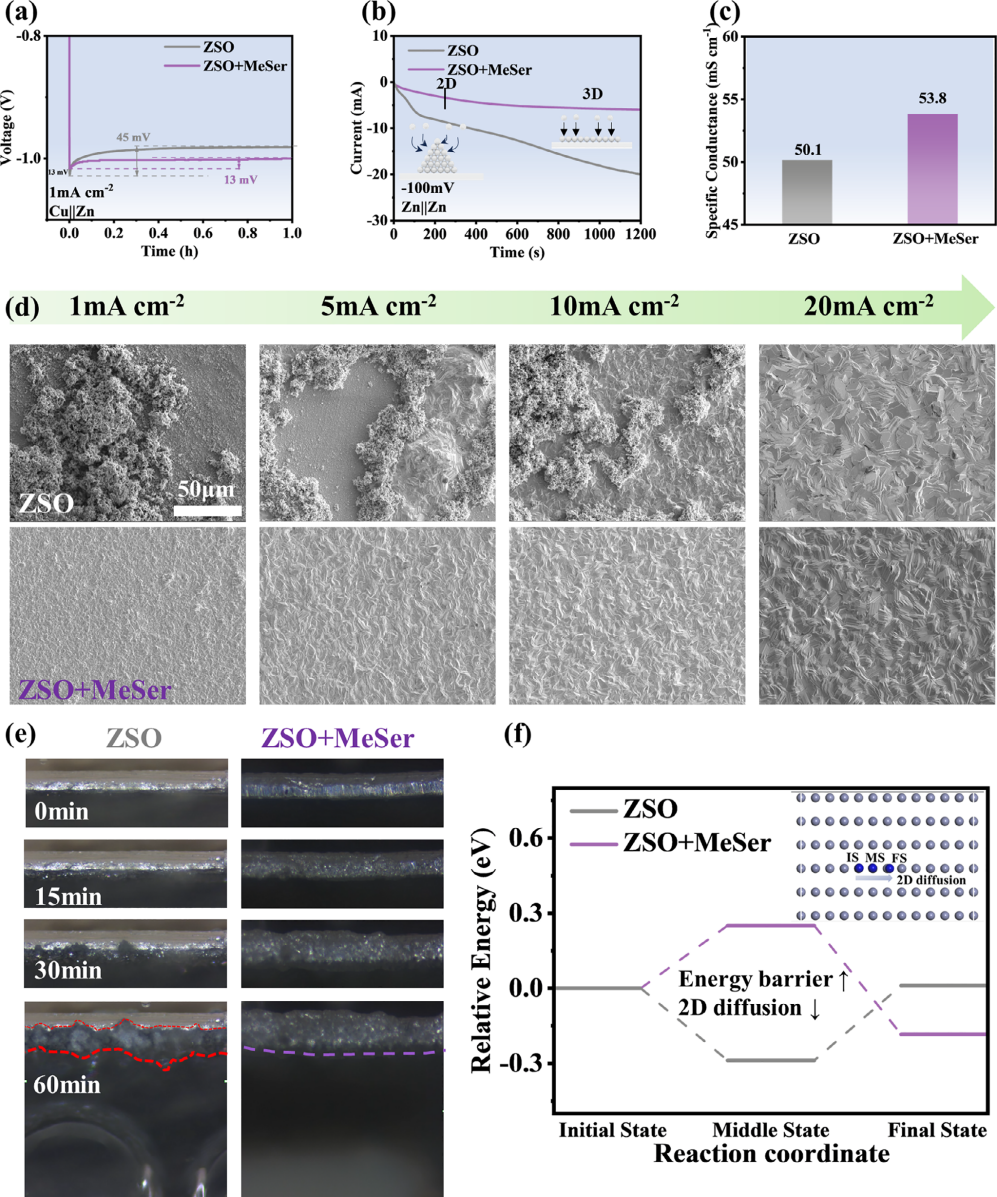
a) Zinc nucleation overpotential in Cu||Zn cells at 1 mA cm^−2^. b) CA curve of Zn||Zn cells at an overpotential of ‐100 mV. c) Conductivity of different electrolytes. d) SEM images of zinc deposits on Cu substrates after 1 h of electrodeposition in different electrolytes. e) In situ optical microscopy of zinc deposition at 10 mA cm^−2^. f) Diffusion energy barrier of Zn^2^⁺ on bare zinc and zinc adsorbed with MeSer^−^.

With the confirmation that MeSer^−^ actively integrates into the EDL, we proceeded to analyze Zn^2^⁺ nucleation and deposition mechanisms. **Figure** **4**a highlights that the optimized EDL exerts a stronger driving force, lowering the zinc nucleation potential in ZSO+MeSer by 13 mV relative to ZSO. More importantly, the difference between nucleation and growth potential in ZSO+MeSer is only 13 mV, significantly smaller than the 45 mV observed in ZSO, indicating faster and more uniform zinc deposition. Since 2D diffusion is a critical factor in zinc dendrite formation, enabling a rapid transition from 2D to 3D diffusion is essential for stabilizing zinc deposition and improving cycle performance. Figure 4b presents CA curves capturing current variation at ‐100 mV. In ZSO electrolyte, Zn^2^⁺ experiences prolonged 2D diffusion without transitioning into 3D diffusion even after 1200 s. In contrast, in ZSO+MeSer, the attractive effect from EDL drives a shift from 2D to 3D diffusion within 200 s, significantly facilitating smoother zinc deposition.^[^ ^34^ ^]^ The conductivity of the electrolyte was directly measured using a conductivity meter (Figure 4c). ZSO+MeSer exhibits higher conductivity, which indicates faster Zn^2^⁺ transport.

Scanning electron microscopy (SEM) and in situ optical microscopy were employed to assess the impact of EDL modification on zinc deposition kinetics. Figure 4d and Figure S8–S12 (Supporting Information) show the morphologies of deposits formed on Cu substrates following 1 h of plating under varying current densities. In ZSO electrolyte, after 1 h of plating at 1 mA cm^−2^, moss‐like zinc deposits emerged on the Cu substrate due to the uneven Zn^2^⁺ deposition and excessive dendrite formation. At 5 mA cm^−2^, zinc deposits in ZSO electrolyte remained incomplete, while at 10 mA cm^−2^, full substrate coverage was achieved due to enhanced deposition forces. At 20 mA cm^−2^, zinc deposits in ZSO displayed a relatively smooth surface. In stark contrast, zinc deposits obtained in ZSO+MeSer electrolyte at 1 mA cm^−2^ were already smooth and free of irregularities, attributable to the rapid deposition process, dominant 3D diffusion, and balanced Zn^2^⁺ concentration distribution. With increasing current density, zinc deposits in ZSO+MeSer maintain their smoothness. These findings confirm that EDL compression facilitates Zn^2^⁺ 3D diffusion, promoting uniform zinc deposition.

In situ optical microscopy enables direct observation of nucleation and deposition dynamics throughout the plating process (Figure 4e). In ZSO electrolyte, after 15 min of deposition, small dendrites begin forming on the zinc anode surface, leading to further dendrite development. After 30 min, corrosion pits emerge, and by 60 min, severe dendrite growth, corrosion damage, and hydrogen bubbles indicate pronounced electrode degradation without EDL adjustments. By contrast, MeSer incorporation significantly inhibits early dendrite formation, ensuring the electrode surface remains smooth even after 30 min of deposition. After 60 min, the electrode retains a compact, uniform coating with no gas bubbles, verifying the success of EDL optimization. Tafel and LSV tests also confirmed that the ZSO+MeSer electrolyte exhibits excellent corrosion resistance and HER suppression performance (Figure S13, Supporting Information). To explore the impact of MeSer on deposition kinetics in greater depth, DFT calculations analyze Zn^2^⁺ diffusion at the interface. Results indicate that Zn^2^⁺ has a negative 2D diffusion energy barrier on bare zinc, confirming spontaneous diffusion. After MeSer^−^ adsorption onto the zinc surface, the diffusion energy barrier shifts to a positive value, indicating its inhibitory effect on 2D diffusion. These atomic‐scale calculations reveal MeSer^−^ adsorption within the EDL as a key determinant influencing Zn^2^⁺ diffusion mechanisms.

**Figure 5:**
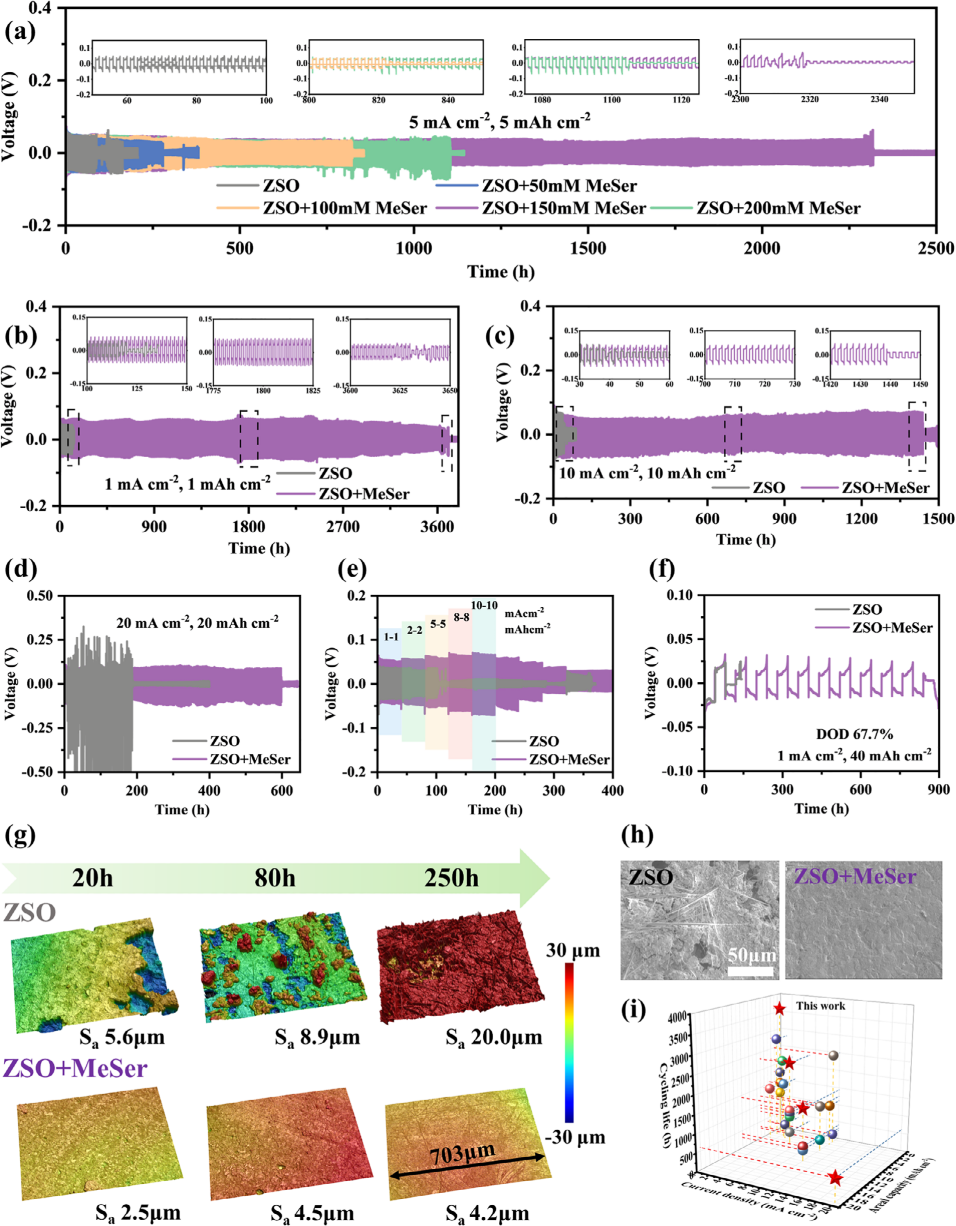
a) Voltage‐time curves of Zn||Zn symmetric cells in electrolytes with different MeSer concentrations at 5 mA cm^−2^ and 5 mA h cm^−2^. Voltage‐time curves of Zn||Zn symmetric cells in different electrolytes at b) 1 mA cm^−2^ and 1 mA h cm^−2^, c) 10 mA cm^−2^ and 10 mA h cm^−2^, d) 20 mA cm^−2^ and 20 mA h cm^−2^, f) 1 mA cm^−2^ and 40 mA h cm^−2^. e) Rate performance of Zn||Zn symmetric cells. g) CLSM images of zinc anodes after cycling in ZSO and ZSO+MeSer electrolytes at 1 mA cm^−2^ and 1 mA h cm^−2^. h) SEM images of zinc anodes after 100 h of cycling at 1 mA cm^−2^ and 1 mA h cm^−2^. i) Comparison of electrochemical performances with previously reported studies.

The enhancement effect of MeSer on battery performance was further verified through cell assembly and testing. Initially, we examined the effect of MeSer concentration on Zn||Zn symmetric cell cycling stability. As shown in **Figure** **5**a, under test conditions of 5 mA cm^−2^ and 5 mA h cm^−2^, adding 50 mM MeSer led to a slight increase in cycling performance. As the MeSer concentration increased to 100 mM, cycling life significantly extended to 1113 h. Further increasing MeSer to 150 mM resulted in a superior cycle life of 2320 h, exceeding most prior studies. However, excessive MeSer content led to performance decline, confirming that ZSO+150 mM MeSer is the optimal electrolyte composition. In CV tests, the introduction of MeSer causes the left inflection point to appear later, which is attributed to its suppression of hydrogen evolution (Figure S14, Supporting Information).^[^ ^35^, ^36^ ^]^ Coulombic efficiency (CE) serves as a key indicator of charge‐discharge effectiveness. Figure S15 (Supporting Information) reveals that Cu||Zn cells using ZSO electrolyte failed after only 110 plating/stripping cycles due to highly unstable zinc deposition and stripping. In contrast, Cu||Zn cells with ZSO+MeSer electrolyte achieved stable cycling for 2000 cycles, with a CE of 99.3%, demonstrating suppression of side reactions and dendrite formation. We continued investigating the cycling behavior of symmetric cells under different testing parameters. At 1 mA cm^−2^ and 1 mA h cm^−2^, the symmetric cell with ZSO+MeSer electrolyte demonstrated an extraordinary cycle life of 3630 h, marking a thirtyfold increase compared to ZSO‐based symmetric cells (Figure 5b). Under high current density conditions of 10 mA cm^−2^ and 10 mA h cm^−2^, the symmetric cell incorporating ZSO+MeSer retained excellent stability for 1438 h. And even under stringent 20 mA cm^−2^ and 20 mA h cm^−2^ conditions, it demonstrated durability for 600 h, significantly outperforming prior research (Figure 5c, d). Conversely, symmetric cells utilizing ZSO electrolyte lasted under 40 h at 10 mA cm^−2^ and 10 mA h cm^−2^, and failed immediately at 20 mA cm^−2^ and 20 mA h cm^−2^. Additionally, Figure 5e highlights the rate capability of symmetric cells with different electrolytes. Compared to ZSO electrolyte, symmetric cells with ZSO+MeSer displayed exceptional voltage stability across diverse current densities. Under deep discharge cycling at 67.7% depth of discharge (DOD), symmetric cells containing MeSer exhibited excellent cycling longevity, recording 840 h—ten times longer than the 82 h seen in ZSO electrolyte‐based symmetric cells (Figure 5f). Furthermore, we investigated the performance of MeSer in zinc trifluoromethanesulfonate (ZnOTF) electrolyte. For ZnOTF, the introduction of MeSer similarly suppresses hydrogen evolution (Figure S16, Supporting Information). MeSer also enhances the rate capability and stability of ZnOTF‐based symmetric cells (Figure S17, Supporting Information). These results demonstrate the versatility of MeSer. Figure 5i presents a comparative analysis of current density, areal capacity, and cycling lifespan, confirming that the additive reported in this study significantly outperforms prior research.^[^ ^20^, ^23^, ^37^, ^38^, ^39^, ^40^, ^41^, ^42^, ^43^, ^44^ ^]^ Furthermore, MeSer offers additional advantages including low cost (< $0.5 per gram), minimal required dosage (∼100 mM), and a straightforward synthesis process.^[^ ^45^ ^]^

To fully understand the stabilizing effect of MeSer on the zinc anode, confocal laser scanning microscopy (CLSM), SEM, X‐ray diffraction (XRD), and X‐ray photoelectron spectroscopy (XPS) were used to characterize the surface morphology and composition of zinc anodes at different cycling durations. As shown in Figure 5g, CLSM provides a direct visualization of the evolution of the anode surface morphology over time. After 20 h of cycling in ZSO, visible corrosion pits appeared on the zinc anode surface, with a Sa (arithmetic mean height) of 5.6 µm. At 80 h, columnar dendrites developed, negatively impacting battery cycling stability, increasing Sa to 8.9 µm. After 250 h of cycling, the zinc anode surface was covered with protrusions of several hundred micrometers in size, with roughness reaching up to 20µm. In contrast, zinc anodes cycled in ZSO+MeSer maintained a smooth, defect‐free surface, even after 250 h of cycling. The Sa values at 20 h, 80 h, and 250 h were measured at 2.5 µm, 4.5 µm, and 4.2 µm, respectively, reinforcing the effectiveness of MeSer in preserving zinc anode stability. SEM imaging was employed to observe the morphology of zinc anodes after various cycling durations (Figure 5h; Figures S18–S20, Supporting Information). Across different magnifications, SEM images reveal that zinc anodes cycled in ZSO+MeSer electrolyte consistently exhibit improved uniform deposition characteristics and enhanced corrosion resistance. In contrast, zinc anodes cycled in ZSO exhibited dendritic structures, glass fiber deposits, and corrosion‐induced pits, demonstrating substantial surface degradation. CLSM and SEM findings confirm that MeSer enables smoother zinc plating and cleaner anode surfaces via EDL modulation. The protons ionized from MeSer facilitate the elimination of ZHS in the electrolyte. To verify the presence of cycling‐induced by‐products, XRD characterization was conducted on zinc anodes after 200 h of cycling in different electrolytes (Figure S21, Supporting Information). XRD spectra revealed that in ZSO electrolyte, zinc anodes exhibited prominent peaks of Zn_4_(OH)_6_SO_4_·3H_2_O and Zn_4_(OH)_6_SO_4_·4H_2_O, confirming considerable ZHS deposition. In contrast, zinc anodes cycled in ZSO+MeSer electrolyte displayed minimal ZHS peaks, strongly supporting the role of MeSer‐derived protons in eliminating ZHS. To further confirm the effectiveness of EDL modulation, we examined whether MeSer undergoes decomposition leading to SEI formation. Figure S22 (Supporting Information) presents XPS spectra from zinc anodes cycled for 200 h in different electrolytes. The Zn 2p and S 2p spectra of zinc anodes cycled in ZSO and ZSO+MeSer were consistent, indicating that MeSer does not decompose to form SEI during cycling.^[^ ^46^ ^]^ These combined XPS and electrochemical results demonstrate that MeSer primarily improves battery performance via EDL regulation, highlighting the profound impact of EDL modulation strategies.

**Figure 6:**
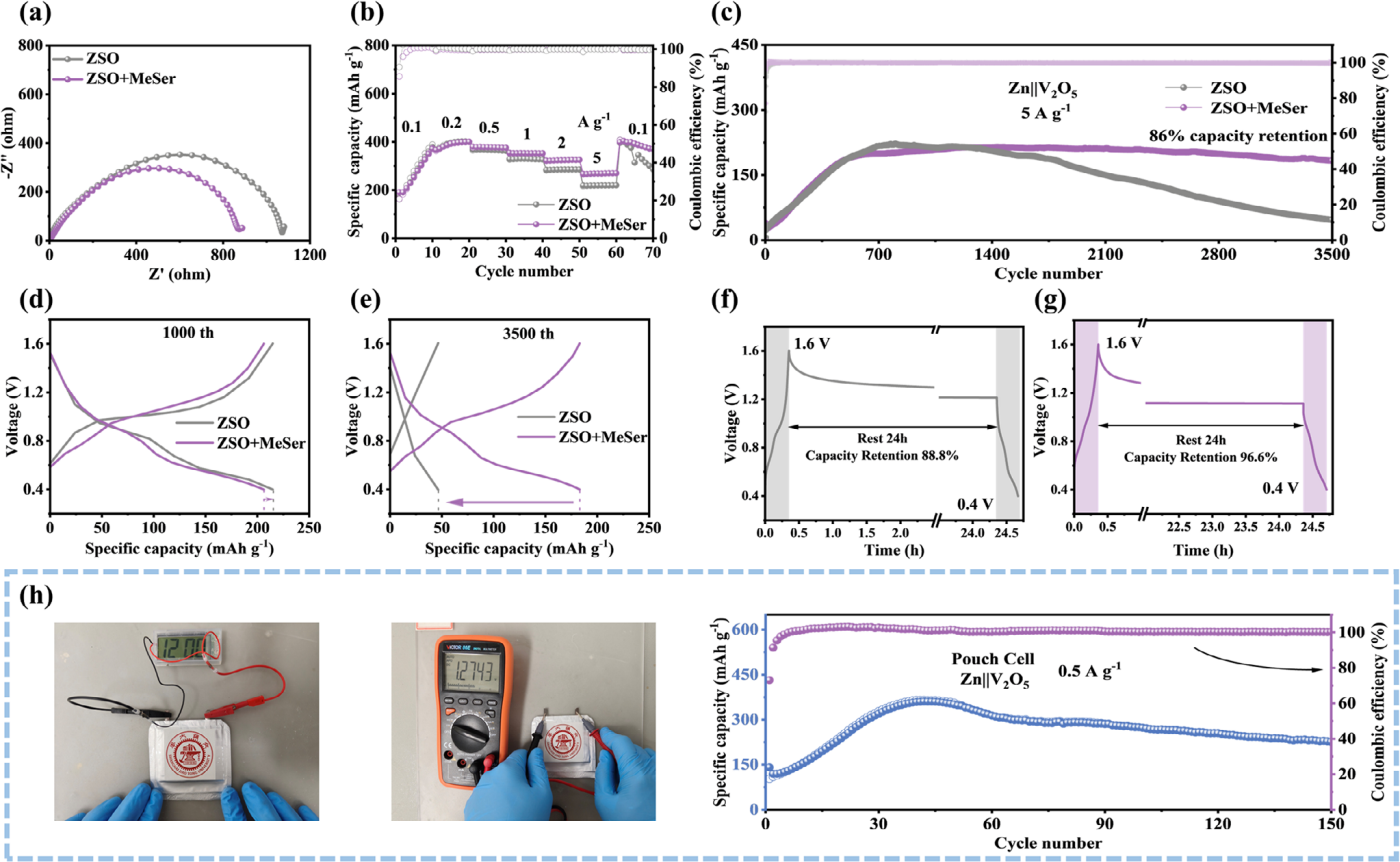
a) EIS of fresh full cells using different electrolytes. b) Rate performance of Zn||V_2_O_5_ full cells in different electrolytes. c) Long‐term cycling performance of Zn||V_2_O_5_ full cells at 5 A g^−1^ in different electrolytes. d, e) Different cycles of charge/discharge profiles of Zn||V_2_O_5_ full cells at 5 A g^−1^ in different electrolytes. Self–discharge test of Zn||V_2_O_5_ full cells based on f) ZSO electrolyte and g) ZSO+MeSer electrolyte. h) Long‐term cycle of the pouch cell.

To explore the practical application potential of ZSO+MeSer electrolyte, a Zn||V_2_O_5_ full cell was assembled, with V_2_O_5_ as the cathode paired with a zinc anode. Figure S23 (Supporting Information) presents the CV curves of Zn||V_2_O_5_ full cells using different electrolytes, indicating that the redox peaks remain largely unchanged, demonstrating that the introduction of the additive does not alter electrochemical responses.^[^ ^47^ ^]^ The reduction in CV curve area may be attributed to the strong adsorption of the MeSer^−^ on the cathode material. DFT calculations showed that the adsorption energy of MeSer^−^ on V_2_O_5_ is significantly greater than that of H_2_O, SO_4_^2^^−^, and Zn^2^⁺ (Figure S24, Supporting Information). EIS measurements of full cells with various electrolytes reveal that the ZSO+MeSer system exhibits a lower charge transfer resistance, signifying accelerated electrochemical reaction kinetics (**Figure** **6**a). The rate performance of full cells was further evaluated at current densities of 0.1 A g^−1^, 0.2 A g^−1^, 0.5 A g^−1^, 1 A g^−1^, 2 A g^−1^, and 5 A g^−1^. Figure 6b compares the rate performance curves, illustrating that Zn||V_2_O_5_ full cells using ZSO+MeSer achieve superior stability and higher specific capacity at high current densities. At 5 A g^−1^, the specific capacity of the full cell with ZSO electrolyte is 219 mA h g^−1^, whereas with ZSO+MeSer, it increases to 270 mA h g^−1^. When the current density is returned to 0.1 A g^−1^, the capacity of full cells with ZSO electrolyte declines rapidly, whereas those with ZSO+MeSer remain significantly more stable. These findings confirm that MeSer enhance electrode reaction kinetics.^[^ ^48^ ^]^ Subsequently, long‐term cycling stability was investigated, as depicted in Figure S25 (Supporting Information), which shows the cycling curves at 1 A g^−1^. Under low current density conditions, Zn||V_2_O_5_ full cells using ZSO+MeSer exhibit significantly improved stability, sustaining capacity over 500 cycles without degradation. In contrast, Zn||V_2_O_5_ full cells with ZSO exhibit pronounced capacity decline after 250 cycles. When the current density increases to 5 A g^−1^, the effect becomes more pronounced. As shown in Figure 6c, after 700 cycles, the capacity of the full cell using ZSO starts to decline continuously, and after 3500 cycles, the specific capacity drops to only 46.52 mA h g^−1^. In contrast, after introducing MeSer, the cycling stability of the full cell at high current densities is significantly improved, with a capacity retention rate of 86% after 3500 cycles and a final specific capacity of 183.07 mA h g^−1^. The long‐term cycling test results indicate that MeSer substantially enhances the cycling stability of full cells under high‐rate conditions, primarily due to its suppression of Zn^2^⁺ 2D diffusion, enabling smooth deposition and reducing by‐product formation.

To further evaluate the durability of full cells with different electrolytes, a self‐discharge performance test was conducted, as shown in Figure 6f,g. The full cell using ZSO retained 88.8% of its capacity after 24 hours of resting, whereas the full cell with ZSO+MeSer maintained an impressive capacity retention of 96.9% after the same duration, further demonstrating the crucial role of MeSer in improving full‐cell durability. Finally, to investigate the commercial potential of MeSer, a pouch cell was fabricated. Figure 6h presents optical images of the pouch cell, its cycling curve, and its ability to power an LED electronic device. The fresh pouch cell exhibited an open‐circuit voltage of approximately 1.3 V, sufficient to drive small electronic appliances. At a current density of 0.5 A g^−1^, the pouch cell achieved a maximum specific capacity of 365 mA h g^−1^ and maintained stable cycling for over 150 cycles. These results confirmed that the MeSer additive remains effective even when scaled to larger electrode areas. Overall, this study introduces a novel additive that holds great promise for advancing the commercialization of AZIBs through a simple EDL regulation strategy.

## 3 Conclusion

This study introduces MeSer as a novel additive incorporated into ZSO electrolyte, markedly improving the electrochemical performance of AZIBs. MeSer ionizes in solution, generating MeSer^−^ and protons. Results indicate that MeSer^−^ does not alter the solvation structure of Zn^2^⁺ but compresses the EDL to reduce internal repulsive forces, thereby enhancing Zn^2^⁺ 3D diffusion and ultimately achieving uniform zinc deposition. Furthermore, desolvation activation energy analysis and DFT calculations reveal that MeSer^−^ within the EDL lowers the desolvation energy barrier of Zn^2^⁺ due to its strong affinity for Zn^2+^, thereby improving the rate capability of AZIBs. Due to the superiority of MeSer as an additive, the Zn||Zn symmetric cell achieves remarkable cycling stability, operating for up to 3630 h under low current conditions and surviving extreme high‐current stress for 600 h. The Zn||V_2_O_5_ full cell at 5 A g^−1^ maintains 86% capacity retention, achieving 183 mA h g^−1^ after 3500 cycles. This study highlights the profound impact of simple EDL compression strategies on electrochemical behavior of AZIBs.

## Conflict of Interest

The authors declare no conflict of interest.

## Author Contributions

X.R performed conceptualization, methodology, software, data curation, investigation, wrote – original draft. S.Z. wrote – review and edited the original draft. F.S. wrote – review and edited the original draft. S.J. performed resources. F.W. wrote – review and edited the original draft. X.Y. wrote – review and edited the original draft. Y.W. performed funding acquisition, ressources, wrote – review & edited the original draft.

## Supporting information



Supporting Information

## Data Availability

The data that support the findings of this study are available from the corresponding author upon reasonable request.
