# Supplementary material for: Anion Induced Electric Double Layer Compression and Desolvation Optimization Enable Long Life Zinc Anodes under High‐Rate
Source: Adv Sci (Weinh). 2025 Sep 23;12(46):e13291. doi: 10.1002/advs.202513291 (PMC12697823; doi:10.1002/advs.202513291)
Supplement: Supplementary file 1 — Supporting Information [file ADVS-12-e13291-s001.docx]

**Supporting Information**

**Anion Induced Electric Double Layer Compression and Desolvation Optimization Enable Long Life Zinc Anodes Under High-Rate**

Xiangyu Ren^a^, Sibo Zhao^a^, Fang Song ^a^, Shenghong Ju^a*^, FuMing Wang^b^, Xiaowei Yang^c^, Yunwen Wu^a*^

^a^School of Material Science and Engineering, Shanghai Jiao Tong University, Shanghai 200240, PR China

^b^Graduate Institute of Applied Science and Technology, National Taiwan University of Science and Technology, Taipei 106, Taiwan

^c^School of Chemistry and Chemical Engineering, Shanghai Jiao Tong University, Shanghai 200240, PR China

*Email: [shenghong.ju@sjtu.edu.cn](mailto:shenghong.ju@sjtu.edu.cn); [yangxw@sjtu.edu.cn](mailto:yangxw@sjtu.edu.cn); tlwuyunwen@sjtu.edu.cn

**Experimental Section**

**Preparation of Electrolytes**

The ZSO electrolytes were prepared by adding ZnSO_4_·7H_2_O (JS0066, HongKong JiSiEnBei International Trade Co., Limited) to deionized water (produced by UKPRO ultrapure water system) to prepare 2M ZSO electrolyte as the baseline electrolyte. Various amounts of DL-O-Methylserine (MeSer) (Adamas life) were dissolved into 2 M ZSO electrolyte to obtain the 50mM ZSO+MeSer, 100mM ZSO+MeSer, 150mM ZSO+MeSer, 200mM ZSO+MeSer and 300mM ZSO+MeSer electrolytes respectively.

**Preparation of V_2_O_5_ Cathode**

1 g of NH_4_VO_3_ powder (Macklin) was put into a crucible and annealed to 400 °C for 2 h. After cooling down, the resultant V_2_O_5_ was collected and fully ground. V_2_O_5_ cathode materials were prepared by mixing V_2_O_5_ powder, carbon black (Energy Chemical) and polyvinylidene fluoride (PVDF, Canrd Technology Co. Ltd) with a mixing weight proportion of 7:2:1 in N-methyl-2-pyrrolidone (NMP, J&K Scientific Ltd). The obtained slurry was coated on graphite foil and the cathode materials were dried at 60 °C under a vacuum for 12 h. The V_2_O_5_ mass loading of the electrode was about 1-2 mg cm^−2^.

**Materials Characterizations**

*In situ* optical microscopy (ZEISS HAL 100), laser microscopy (VK-X3000) and SEM (RISE-MAGNA) were employed to observe the morphology evolution of Zn anodes during the cycling process. XRD (D-POWER, GKINST Co., LTD.) was employed to monitor the changes in crystal plane of Zn deposits under different electrolytes. Confocal microscopic Raman spectrometer (Renishaw in Via Qontor, 532 nm, 28 mW) and Fourier transform infrared spectroscopy (PerkinElmer Spectrum 100) were used to characterize the electrolyte. The electron screening effect in electrolytes was investigated via nuclear magnetic resonance (NMR) spectroscopy (Bruker AVANCE III 600 MHz). *In situ* Raman was characterized by Raman spectrometer (Lab RAM Solei) and electrochemical workstation.

**Cell assembly**

Zn||Zn (Zn anode with Φ=10 mm), Zn||Cu (Zn anode with Φ=10 mm, Cu electrode with Φ=10 mm) and Zn||V_2_O_5_ (Zn anode with Φ=16 mm, V_2_O_5_ cathode with Φ=12 mm) cells were assembled using glass fiber as the separator (Φ=16mm) in CR2032 type battery shells. The thickness of the Zn anode used in this study is 100 μm, which corresponds to a capacity of about 58.8 mA h cm^−2^. The electrolyte volume of each symmetric cell was precisely controlled at 150 μL. Amount of electrolyte added to each full cell was fixed at 250 μl. Pipette was purchased from Bioland Biotechnology Co. Ltd.

**Electrochemical test**

CV (1 mV s^−1^; -0.3 V-0.8 V), CP tests were conducted on Zn||Cu cells employing a Corrtest electrochemical workstation. The chronoamperometry tests were measured based on the Zn//Zn coin cell at a fixed overpotential of -100 mV. LSV was tested from -0.95 V to -1.45 V at 1 mV s^-1^, with Zn foil as working electrode and platinum (Pt) plate as counter electrode, the reference electrode used was Ag/AgCl. Electric double layer capacitance (EDLC) measurements for CV curves of Zn//Zn symmetric cells were taken at a scan rate of 2-10 mV s^-1^between -15 and 15 mV and calculated through the equation C = i/v (C, capacitance; i, current; the value of i was determined by taking half of the current difference between positive and negative scan under each scanning rate). In situ electrochemical quartz crystal microbalance with dissipation monitoring (EQCM) measurements were carried out by a Q-sense Analyzer (Biolin Scientific AB) using Cu-coated quartz crystals with a fundamental resonance frequency of 5 MHz. EQCM was performed under CV testing using a three-electrode system with an Au quartz crystal resonator as the working electrode, Pt wire as the counter electrode, Hg/HgO as the reference electrode, and 0.2 M Na_2_SO_4_ with or without MeSer as the electrolyte.

CV (1 mV s^−1^; 0.2 V-1.6 V) test of Zn|| V_2_O_5_ full cells employed an electrochemical workstation (Corrtest). The galvanostatic discharge-charge tests were performed on a NEWARE battery test system (CT-ZWJPLUS-4S-T-1U). For the self-discharge test of the full battery, it was first activated at 0.1 A g^-1^ for 10 cycles, followed by 10 cycles at 1 A g^-1^, and then allowed to stand for 24 h for the self-discharge test. The Electrochemical Impedance Spectroscopy (EIS, 0.01-10^5^ Hz) of Zn||Zn symmetric cells and Zn||V_2_O_5_ full cells were conducted on an electrochemical workstation (Corrtest) with amplitude of 5 mV.

**Density functional theory (DFT) calculations**

All the density functional theory (DFT) calculations were done via the Vienna Ab initio Simulation Package (VASP)^[1]^. The generalized gradient approximation (GGA) with the exchange-correlation functional Perdew-Burke-Ernzerhof (PBE) was used^[2]^. A cutoff energy of 520 eV was set for the exchange and correction. The convergence criteria with forces on atoms and energy residual were smaller than 0.05 eV/Å and 10^-5^ eV, respectively. Initially, the structure of each crystal was optimized, followed by the construction of the corresponding unit cell box. A 15Å vacuum layer was included above the crystal plane to mitigate the influence of repetitive structures on subsequent calculations. with a k-point set of 3×3×1. DFT-D3 correction was applied to address intermolecular dispersion forces^[3]^.

The calculation method for adsorption energy (E) is as follows:

$$E=E_{ab}-E_{a}-E_{b}$$

Here, E_ab_ represents the energy of the system after adsorption, while E_a_ and E_b_ denote the energies of the two components before adsorption, respectively.

**Molecular dynamics (MD) simulation**

All MD simulation for solvation structure investigation was conducted with the Forcite module with the COMPASS Ⅲ force field. We constructed the simulated solution box and made this box energy minimum. The box dimensions were set at 26.3 Å × 26.3 Å × 26.3 Å, employing periodic boundary conditions in all three axes. Each simulation cell consisted of 510 H_2_O molecules, 25 ZnSO_4_ molecules, and 10 BED. Geometry optimization was performed on the model to obtain the initial structure. Following that, the solution models were equilibrated for a 400 ps system duration under constant pressure and temperature conditions. The time step was set as 1.0 fs. Finally, radial distribution function (RDF) and coordination number (CN) were analysis on the final trajectory to examine intermolecular interactions between different atoms.


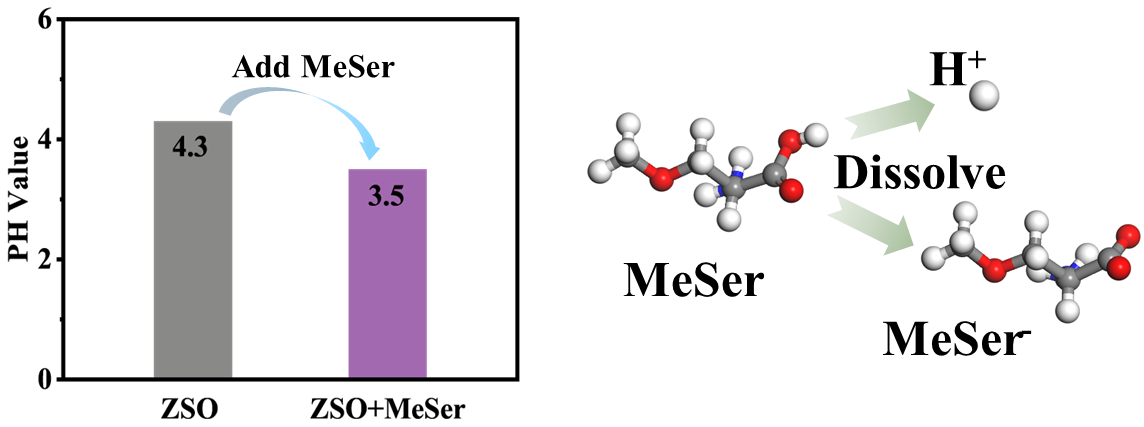


Figure S1. The effect of MeSer on the pH of ZSO electrolyte and a schematic diagram of MeSer ionization in solution.


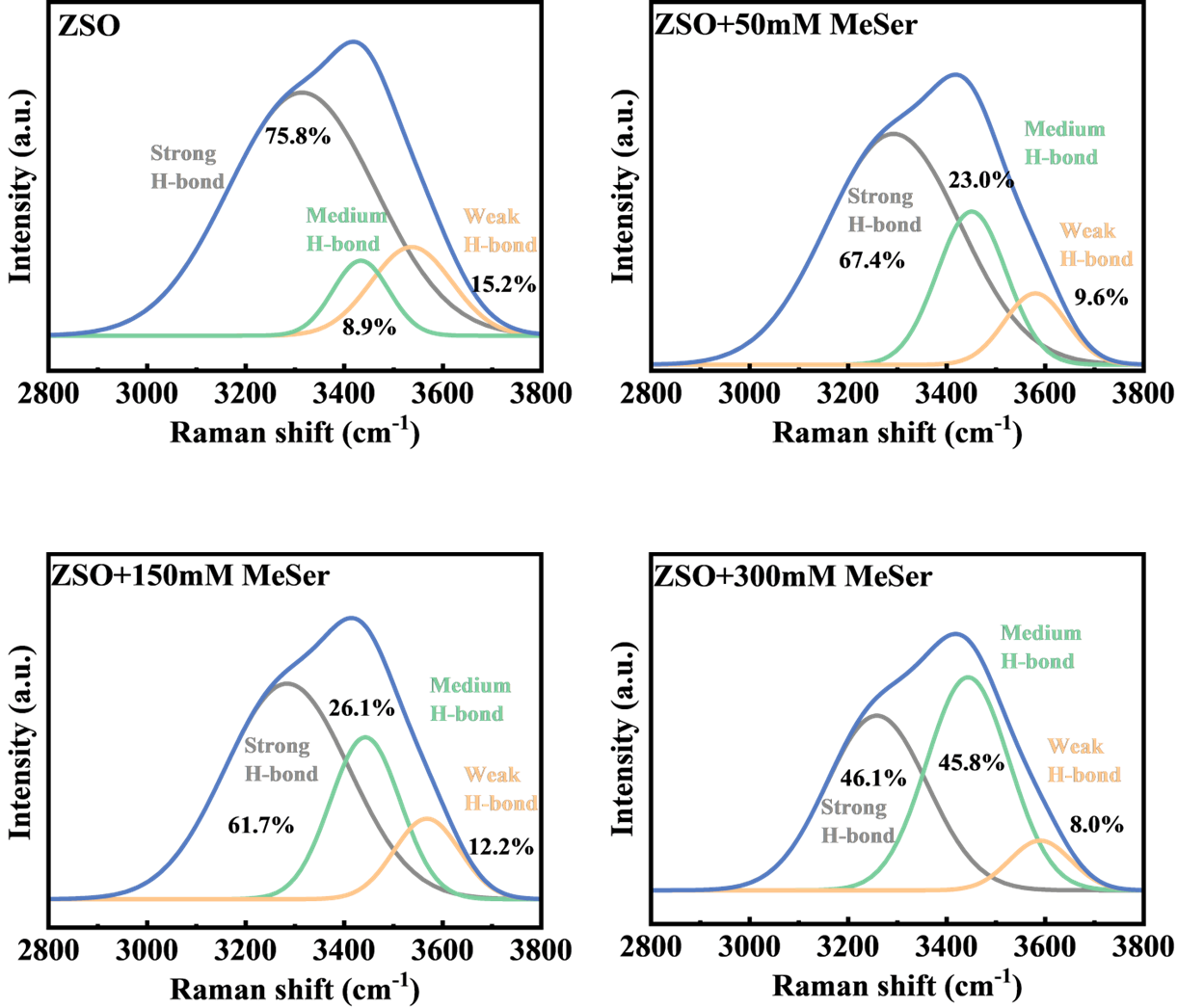


Figure S2. Raman spectra of O-H bond in ZSO electrolytes, ZSO+50mM MeSer electrolytes, ZSO+150mM MeSer electrolytes and ZSO+300mM MeSer electrolytes.


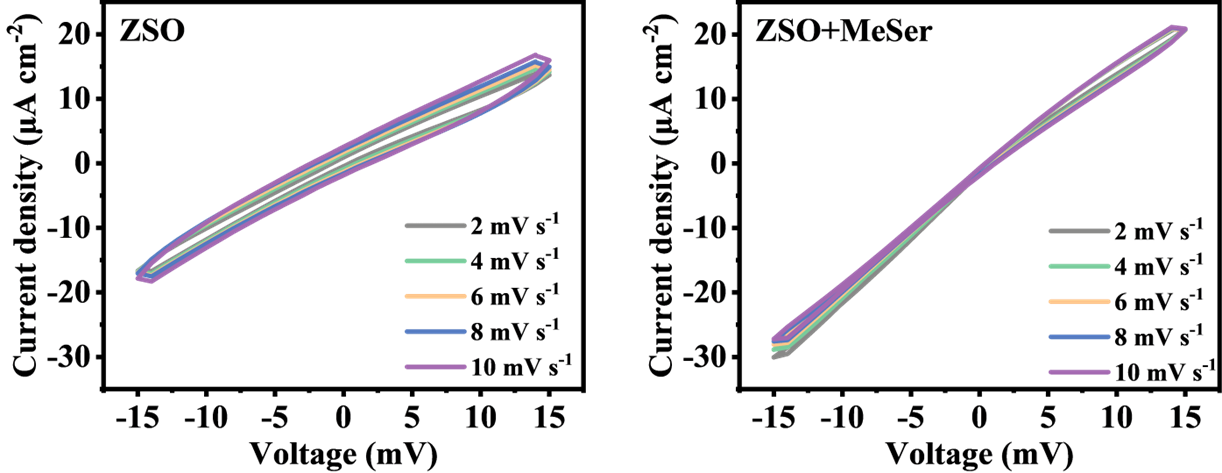


Figure S3. CV curves of symmetric cells at different scan rates within the range of -15 mV to 15 mV.


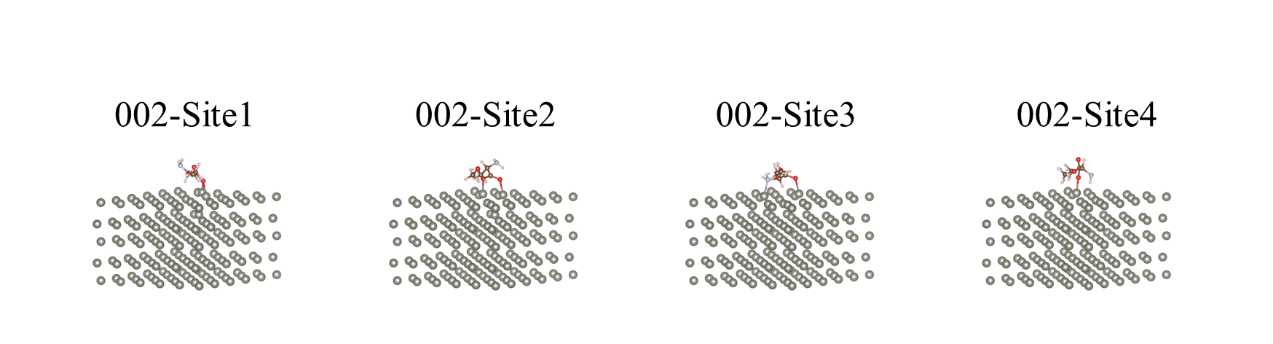


| **Structure** | **Adsorption energy** |
| --- | --- |
| 002-Site1 | -2.205eV |
| 002-Site2 | -2.759eV |
| 002-Site3 | -2.699eV |
| 002-Site4 | -2.002eV |

Figure S4. To determine the optimal adsorption configuration, the adsorption energies of MeSer⁻ on the (002) crystal plane were calculated for different configurations.


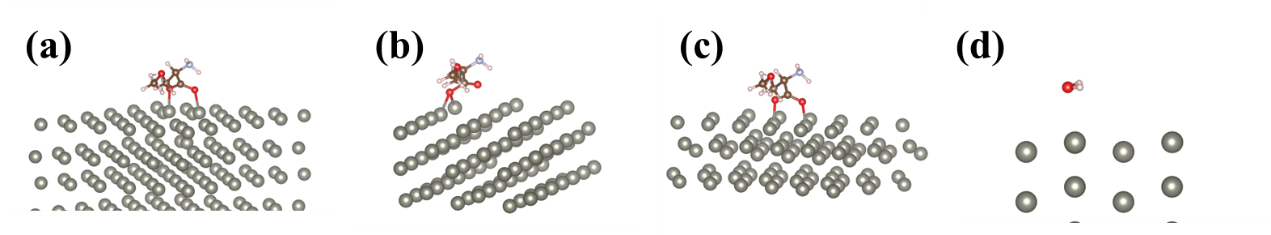


| **Structure** | **Adsorption energy** |
| --- | --- |
| MeSer^-^ on 002 | -2.759eV |
| MeSer^-^ on 100 | -4.298eV |
| MeSer^-^ on 101 | -3.054eV |
| H_2_O on 100 | -0.107eV |

Figure S5. Adsorption energies of MeSer⁻ on zinc surfaces: (a) (002), (b) (100), and (c) (101), along with (d) the adsorption energy of H_2_O on the (100) surface.


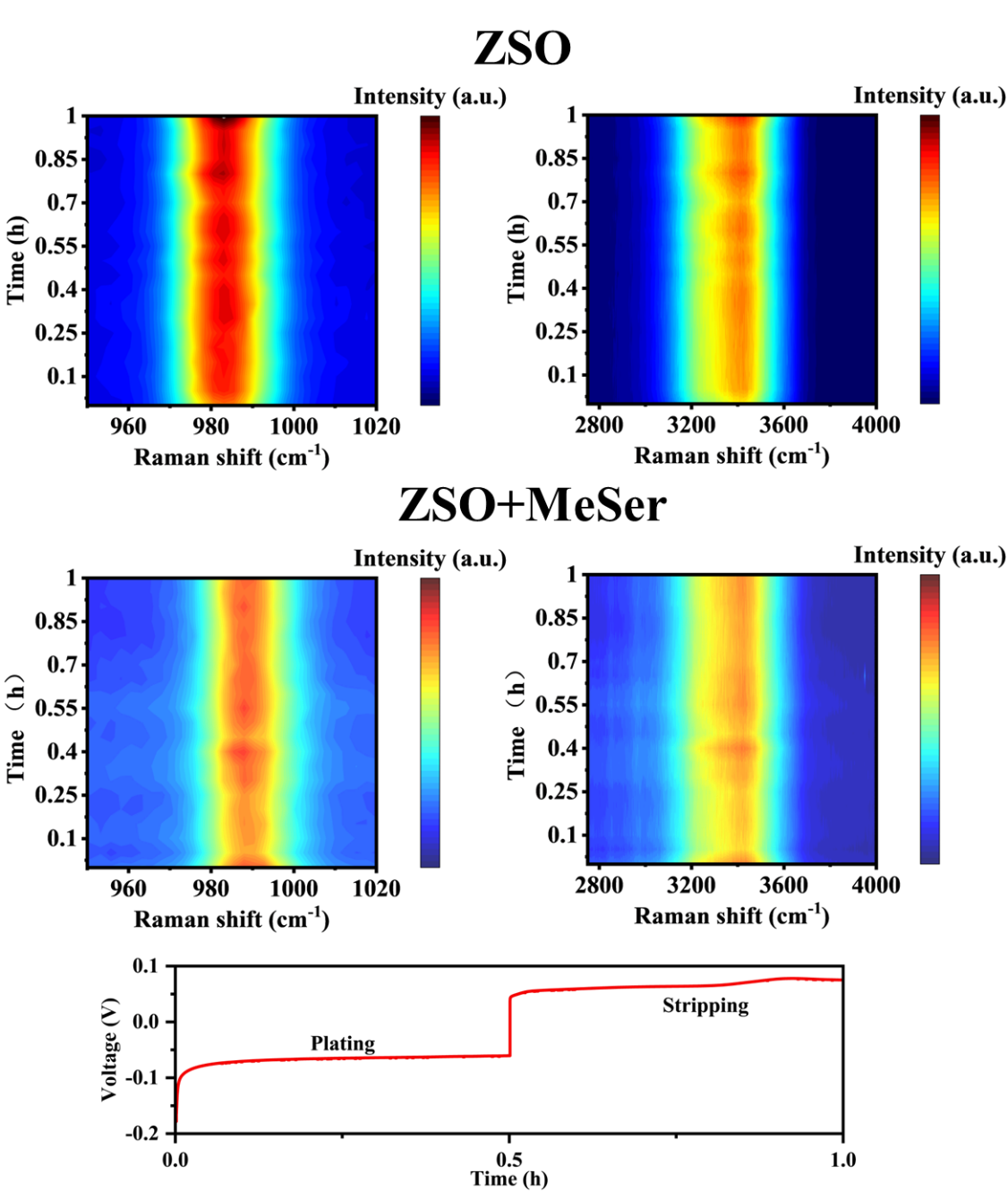


Figure S6. 2D In situ Raman spectroscopy of the anode during plating/stripping in Zn||Zn symmetric cells at different electrolytes.


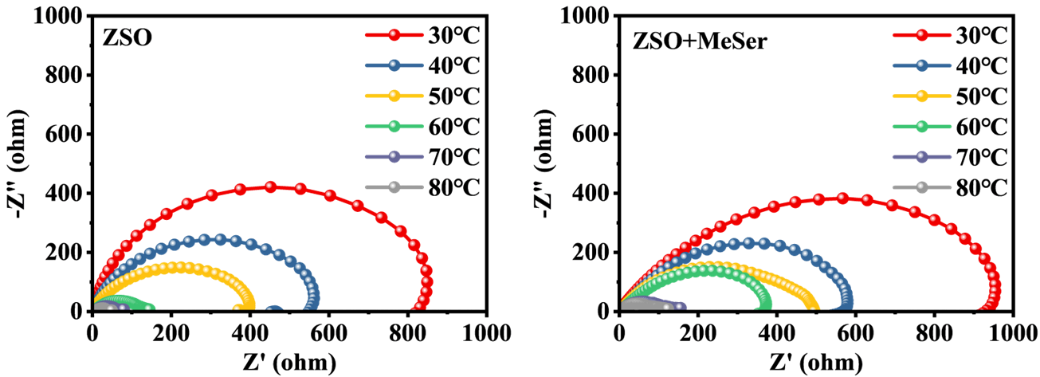


Figure S7. EIS curves of different electrolytes in the temperature range of 30°C to 80°C.


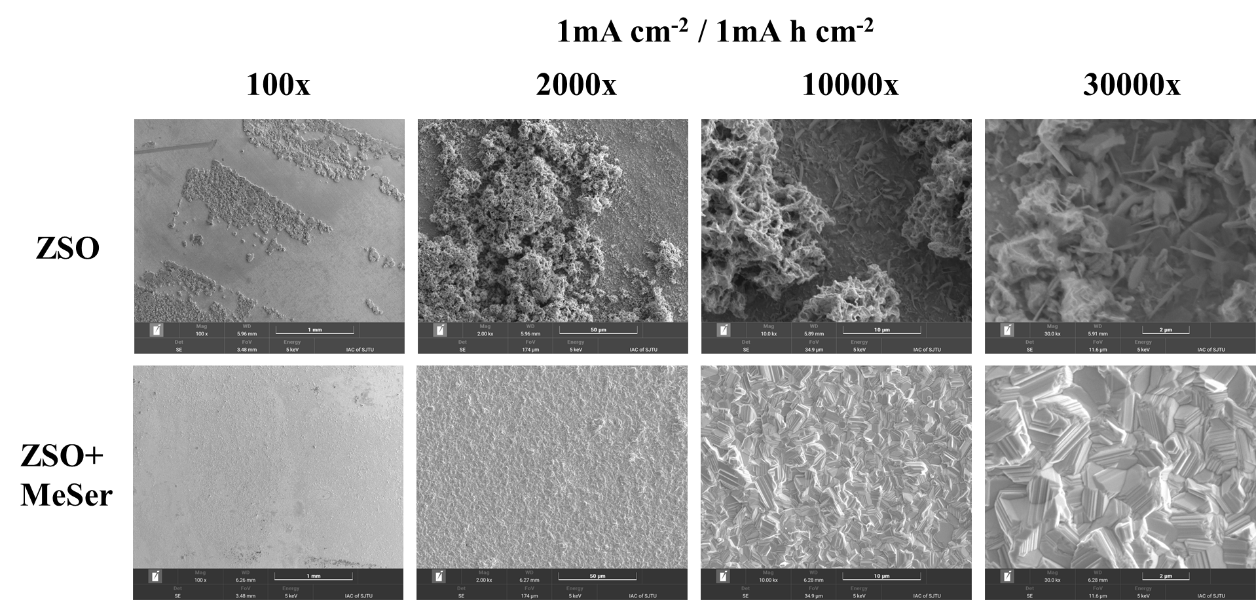


Figure S8. Morphology of Zn deposits on Cu substrates using different electrolytes at 1 mA cm^-2^ and 1 mA h cm^-2^.


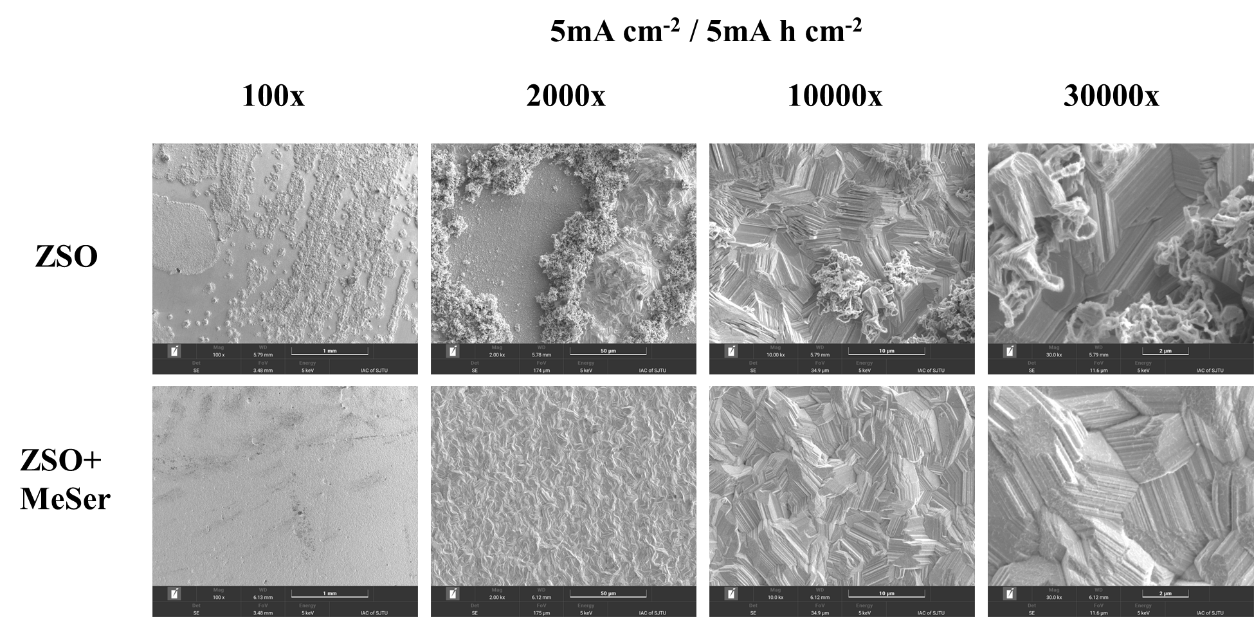


Figure S9. Morphology of Zn deposits on Cu substrates using different electrolytes at 5 mA cm^-2^ and 5 mA h cm^-2^.


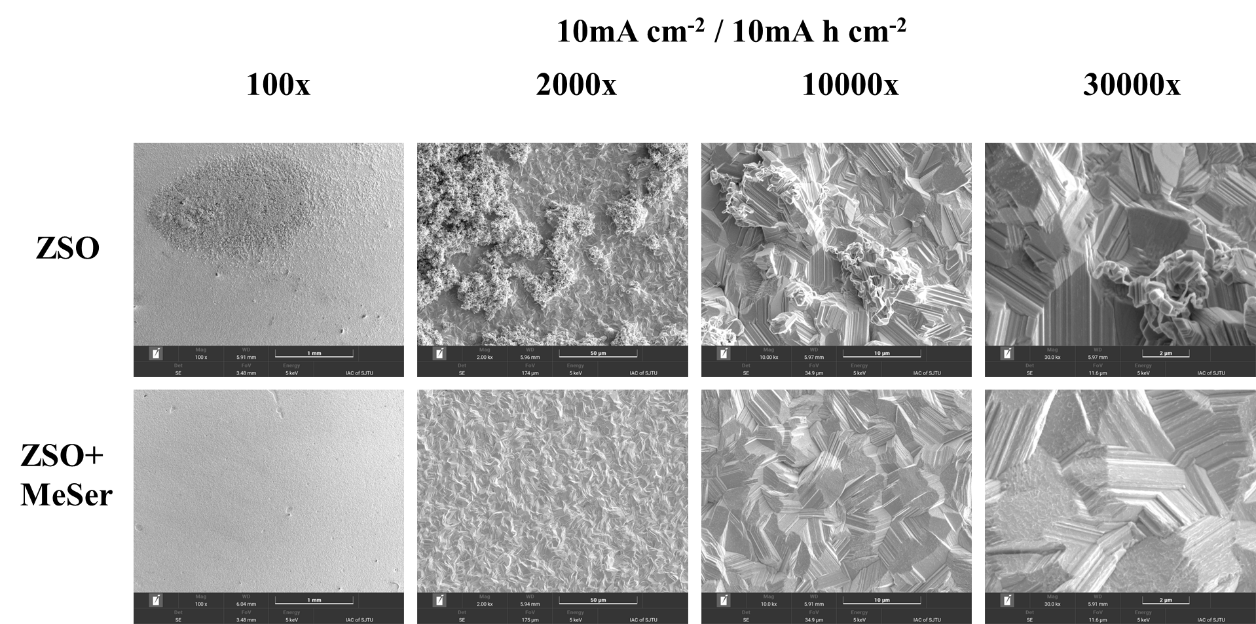


Figure S10. Morphology of Zn deposits on Cu substrates using different electrolytes at 10 mA cm^-2^ and 10 mA h cm^-2^.


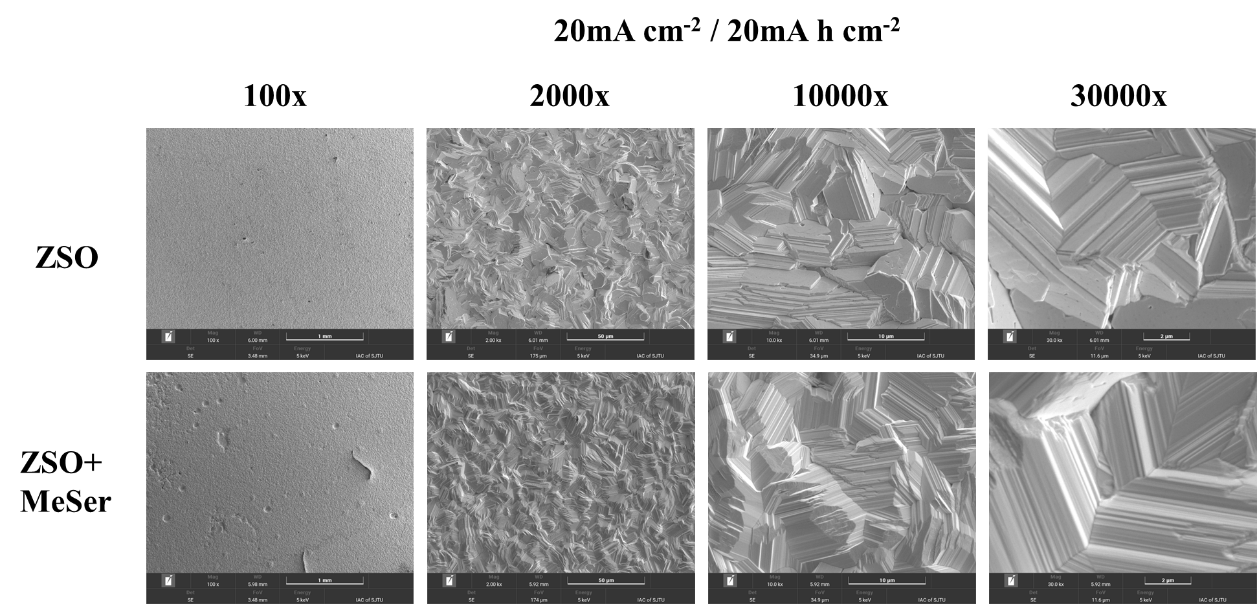


Figure S11. Morphology of Zn deposits on Cu substrates using different electrolytes at 20 mA cm^-2^ and 20 mA h cm^-2^.


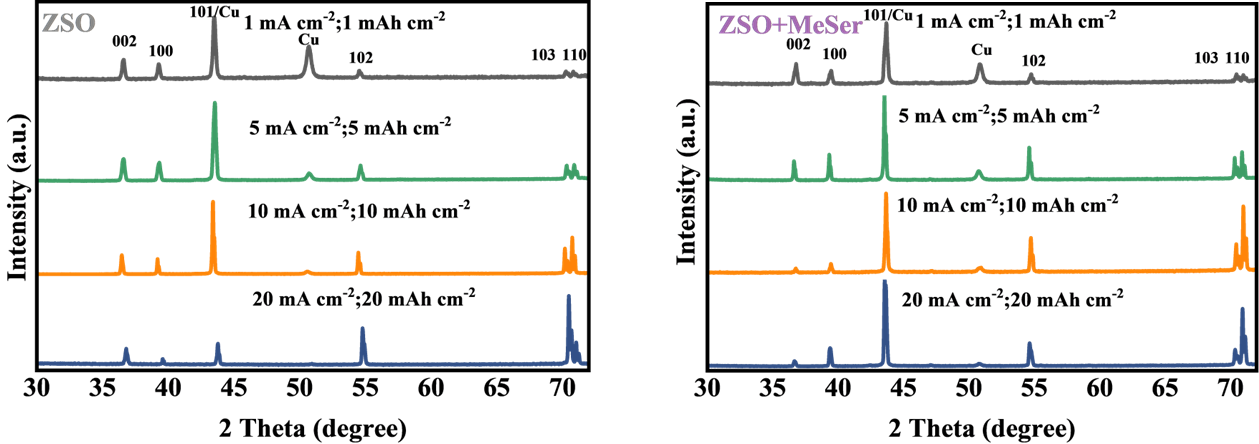


Figure S12. XRD patterns of Zn deposits on Cu substrates using different electrolytes.


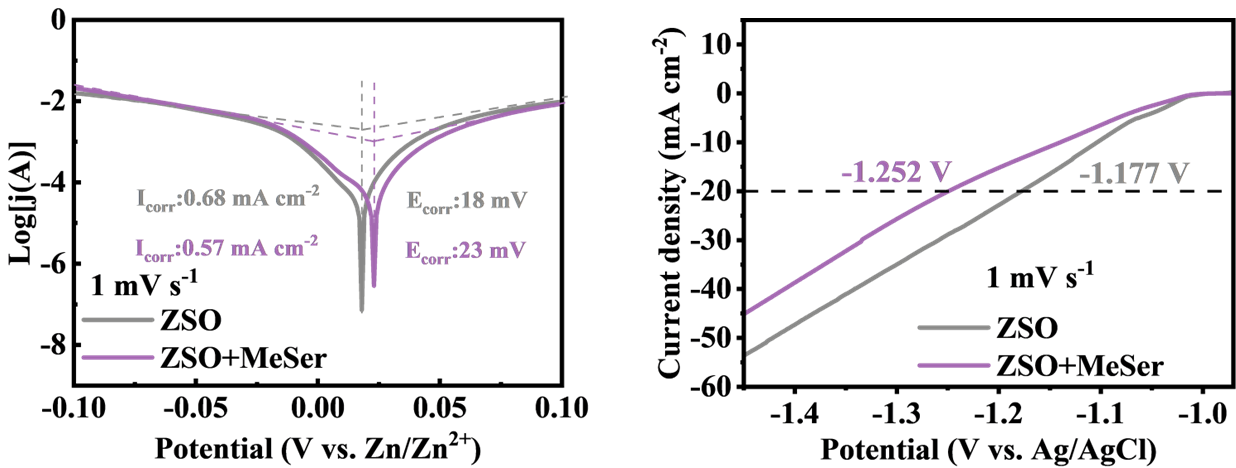


Figure S13. Tafel and LSV tests of zinc in different Electrolyte.


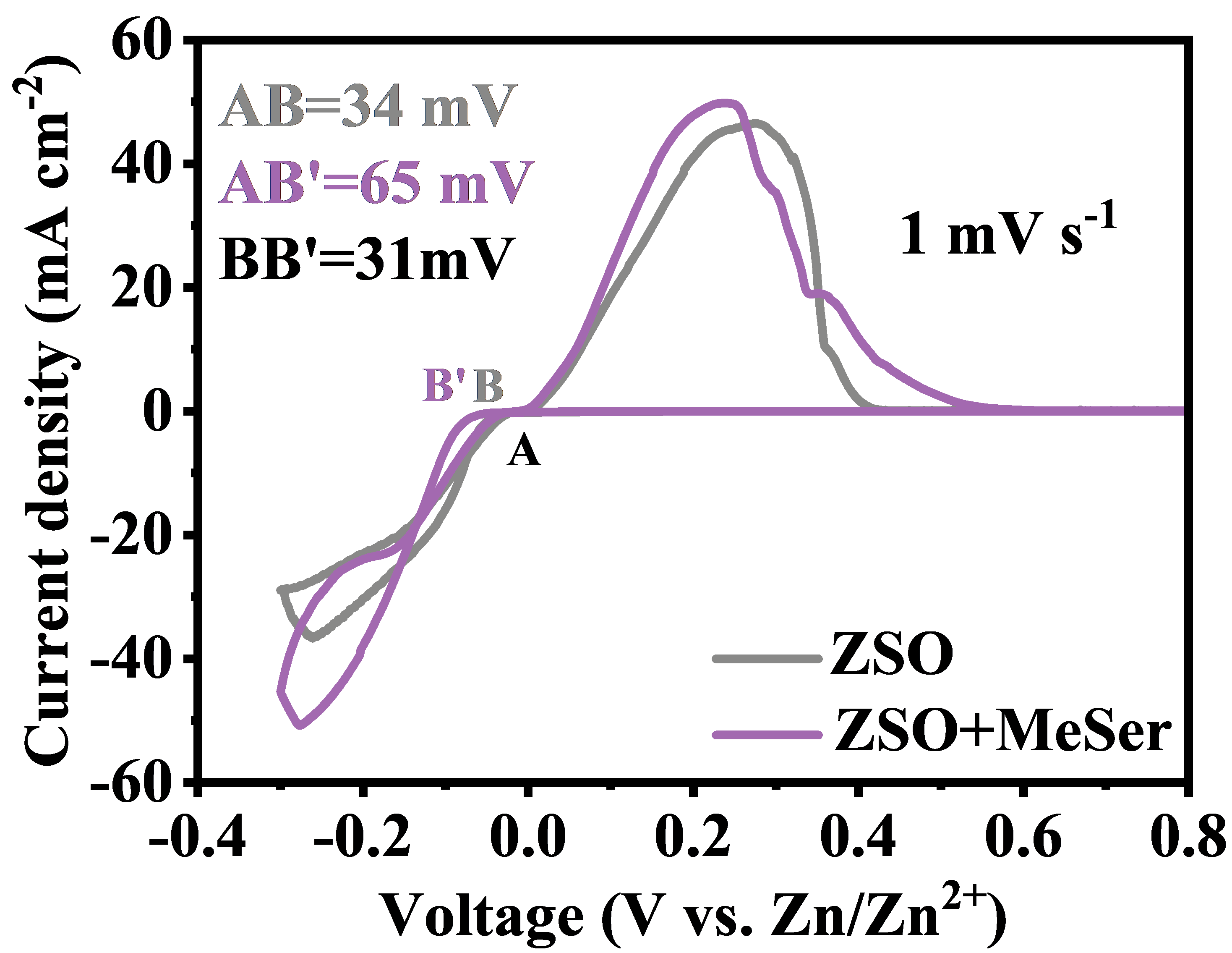


Figure S14. CV curves of Zn||Cu cells in different electrolytes.

Figure S15. CE of Zn||Cu cells in different electrolytes.

**
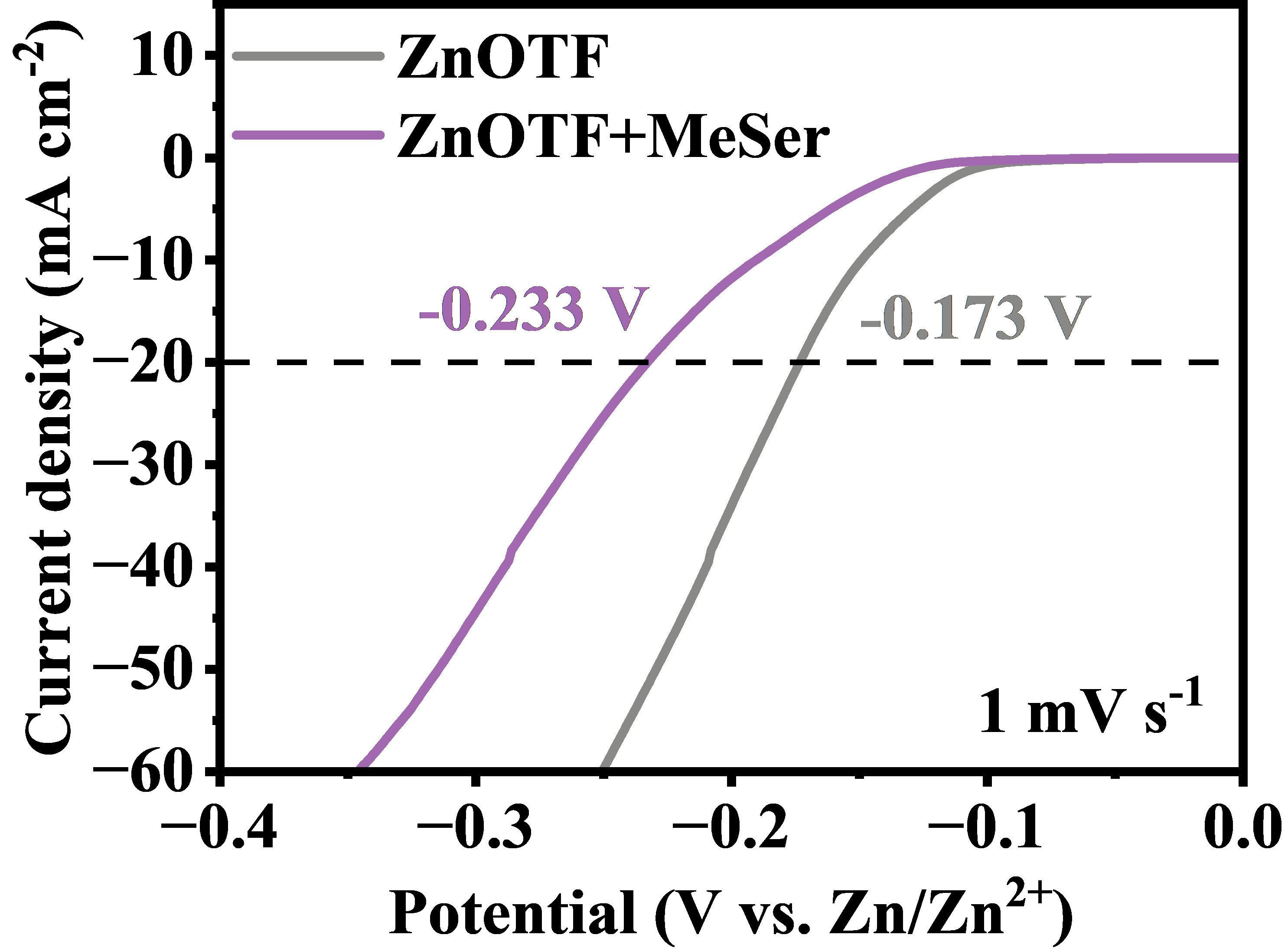
**

Figure S16. Effect of MeSer Addition on HER in ZnOTF Electrolyte.


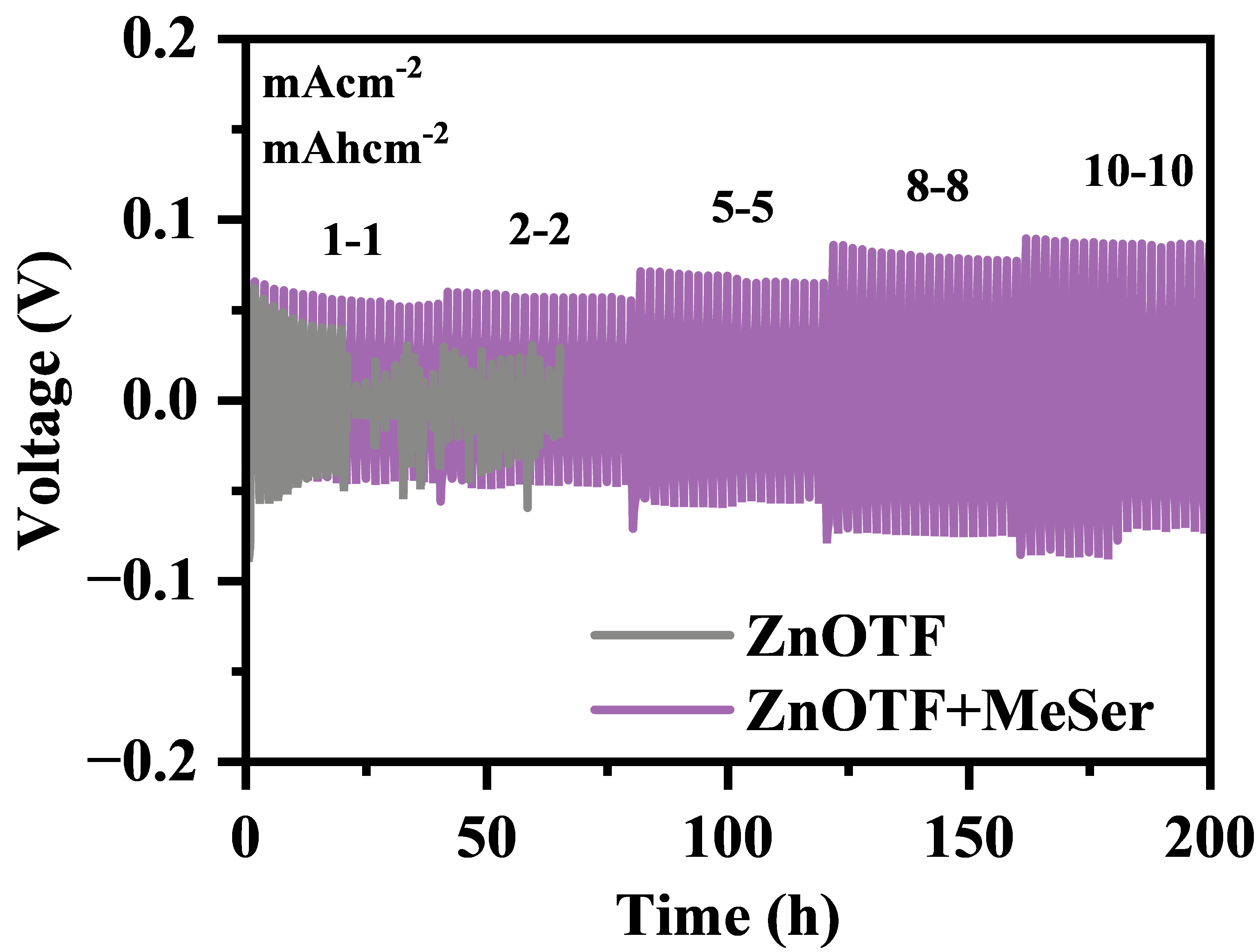


Figure S17. Effect of MeSer on rate performance of symmetric cells in ZnOTF electrolyte.


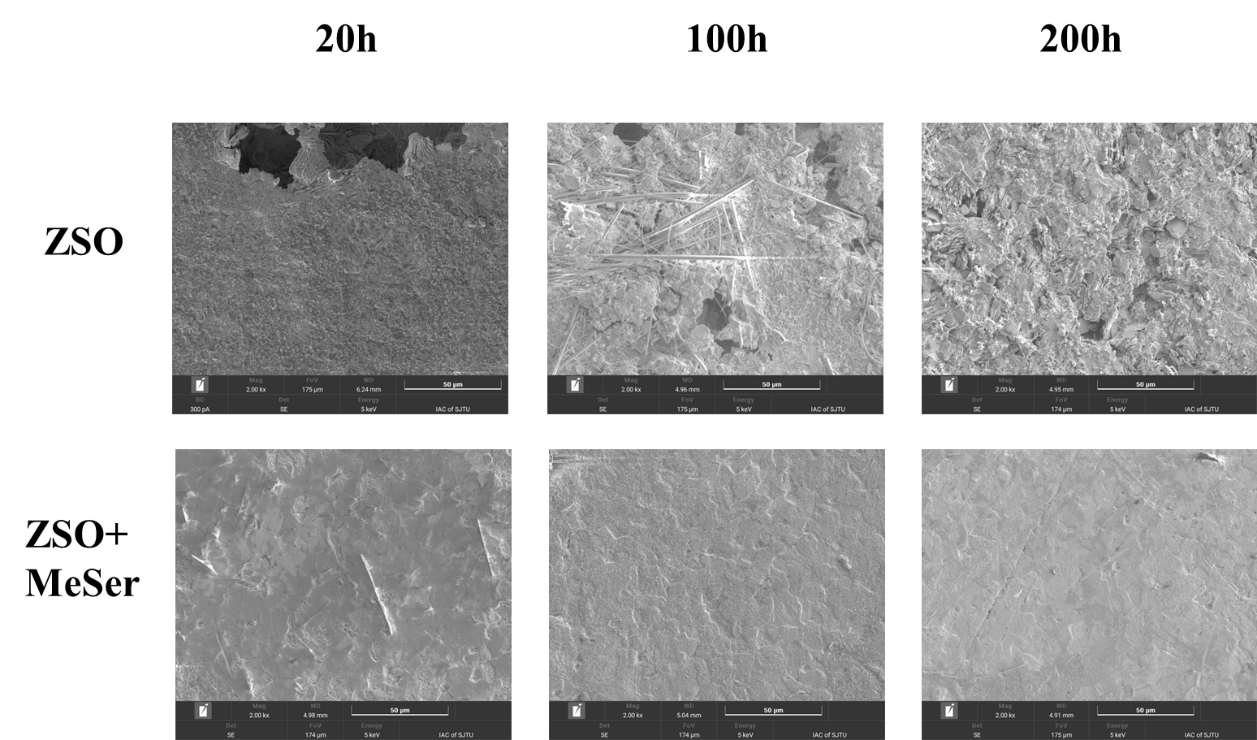


Figure S18. 2000× SEM images of zinc anodes cycled for different durations in various electrolytes.


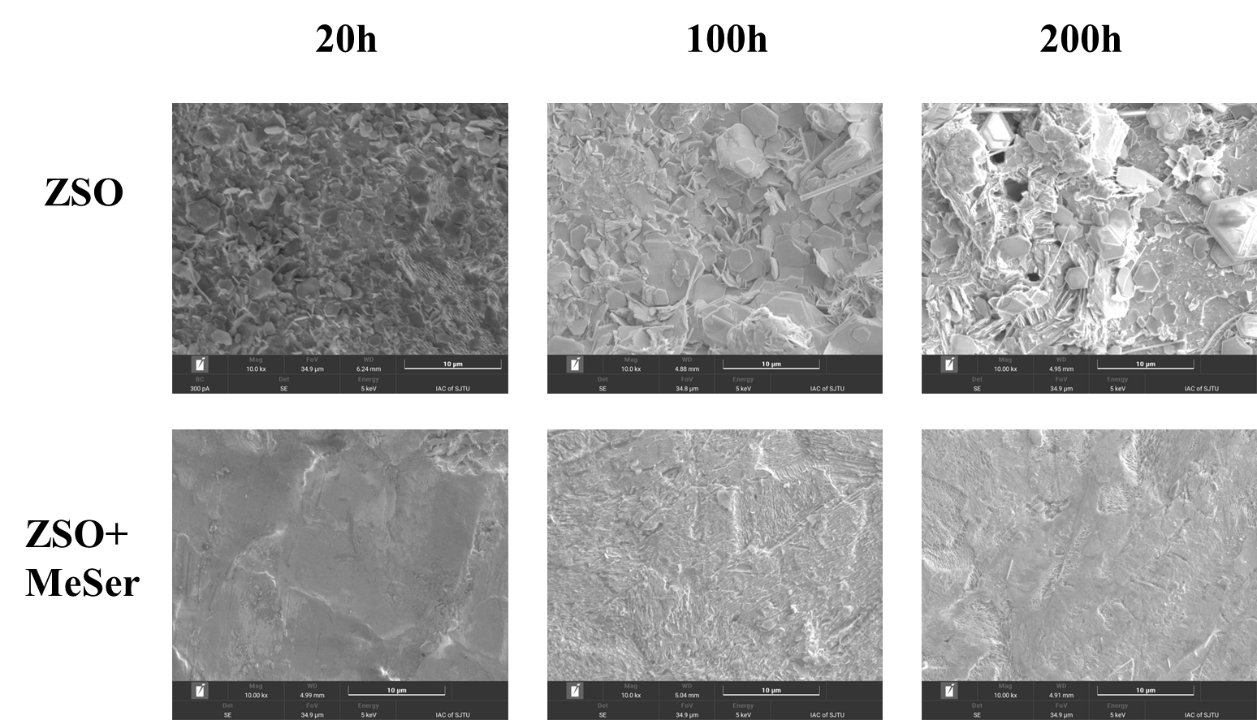


Figure S19. 10000× SEM images of zinc anodes cycled for different durations in various electrolytes.


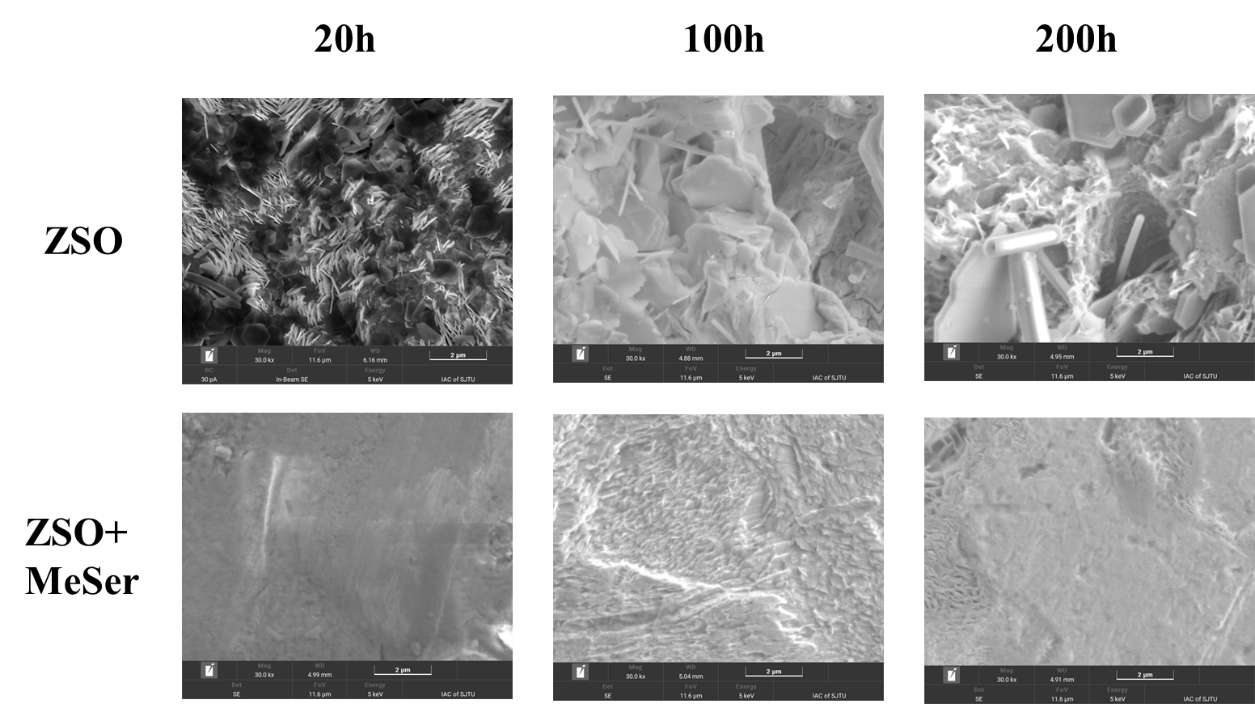


Figure S20. 30000× SEM images of zinc anodes cycled for different durations in various electrolytes.


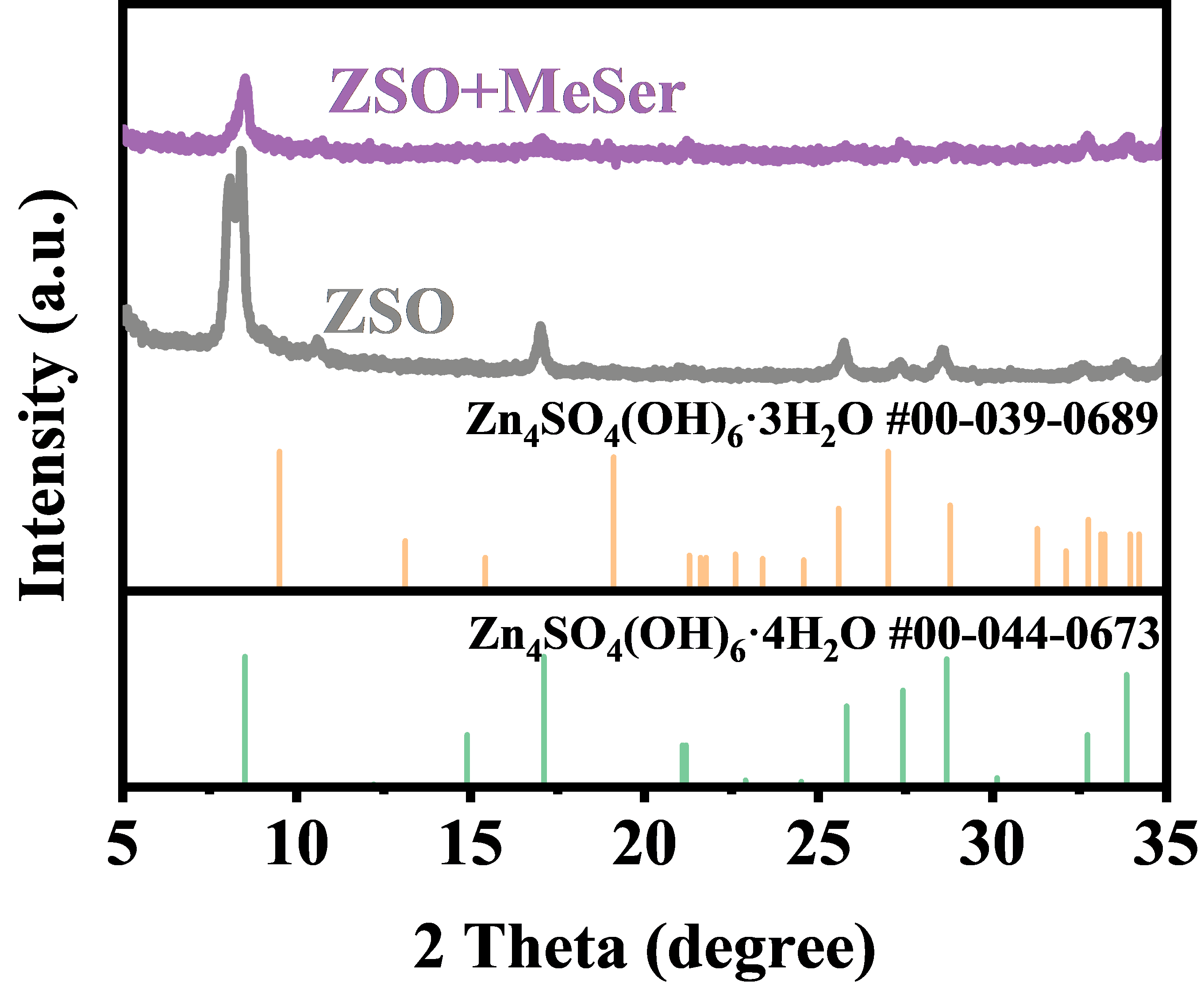


Figure S21. XRD patterns of zinc anodes after 200 h of cycling in different electrolytes under 1 mA cm^-2^ and 1 mA h cm^-2^.


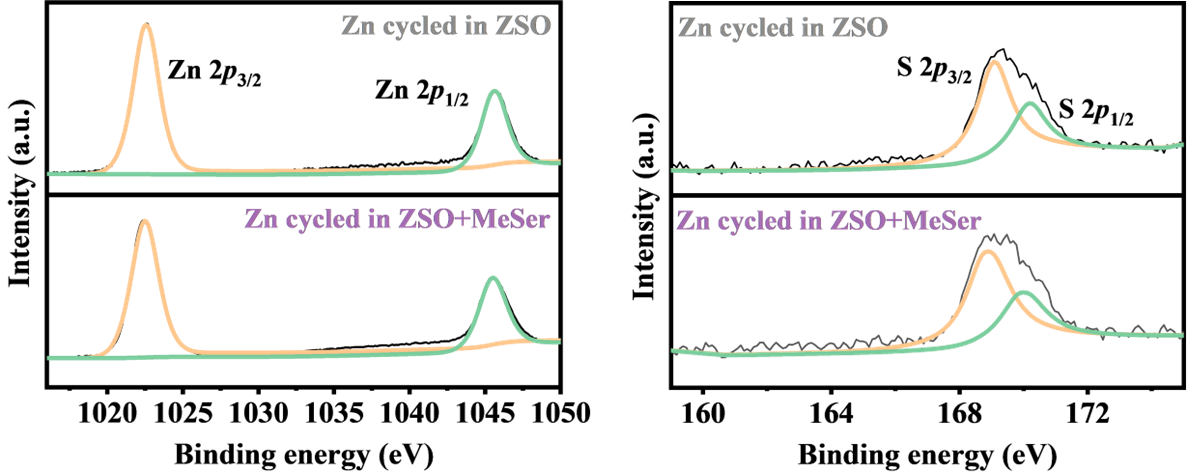


Figure S22. The Zn 2p and S 2p XPS spectra of zinc anodes after 200 h of cycling in different electrolytes under 1 mA cm^-2^ and 1 mA h cm^-2^.


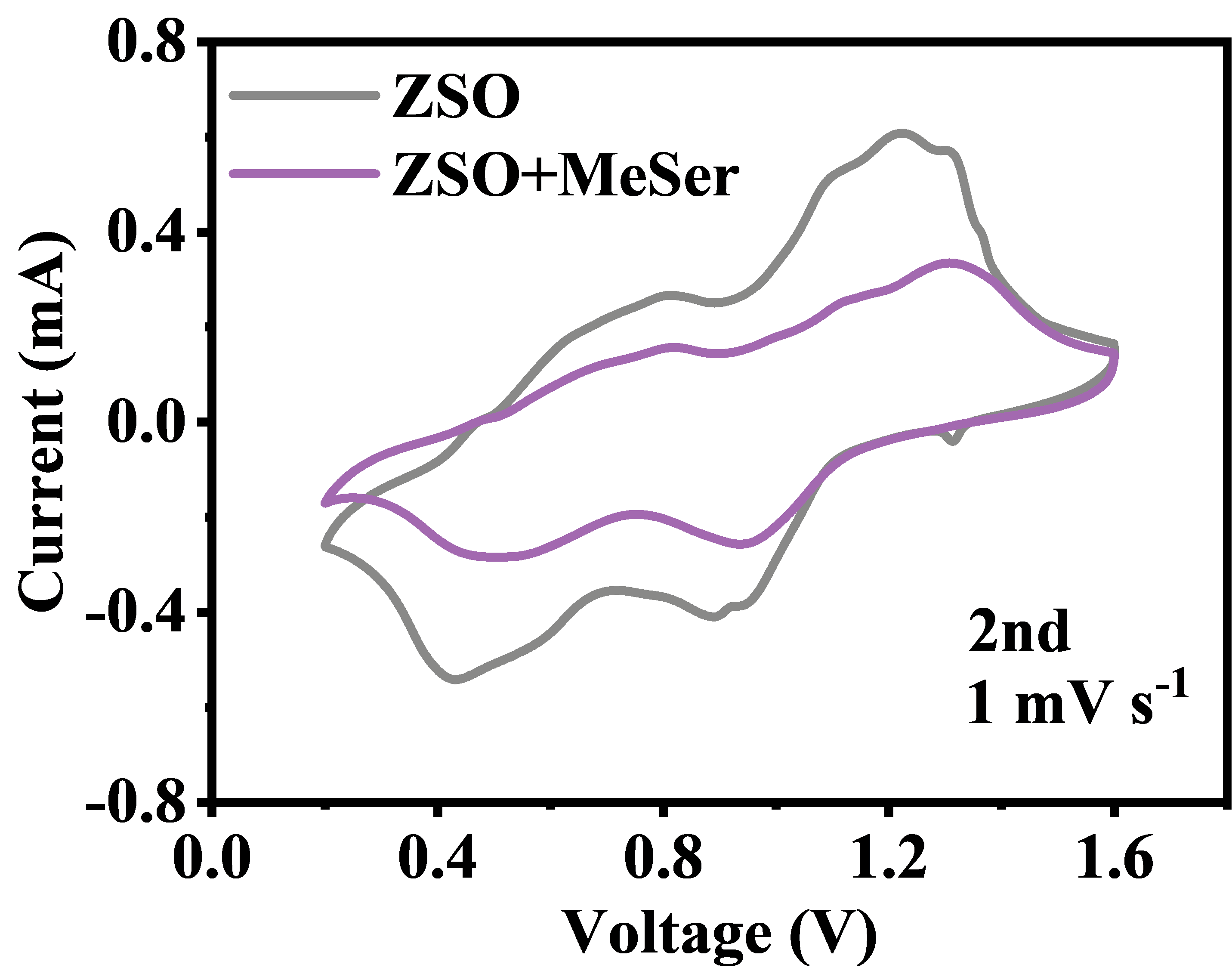


Figure S23. CV curves of Zn||V_2_O_5_ full cells in different electrolytes.


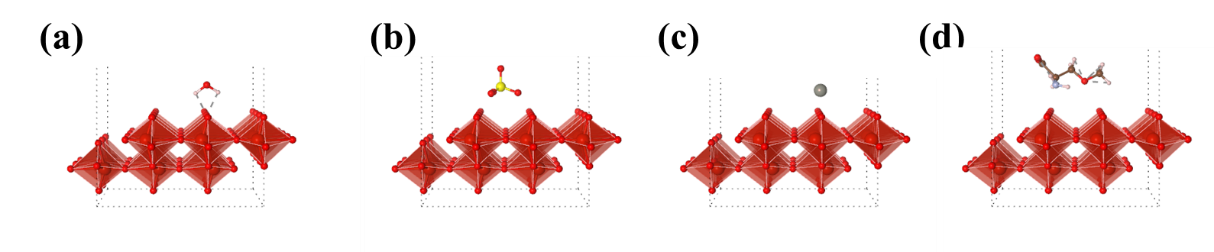


| **Structure** | **Adsorption energy** |
| --- | --- |
| H_2_O on V_2_O_5_ | -0.36eV |
| SO_4_^2-^ on V_2_O_5_ | -0.55eV |
| Zn^2+^ on V_2_O_5_ | -1.02eV |
| MeSer^-^ on V_2_O_5_ | -3.13eV |

Figure S24. Adsorption energies of H_2_O(a), SO_4_^2-^(b), Zn^2+^(c) and MeSer⁻(d) on V_2_O_5_ (001) surfaces.


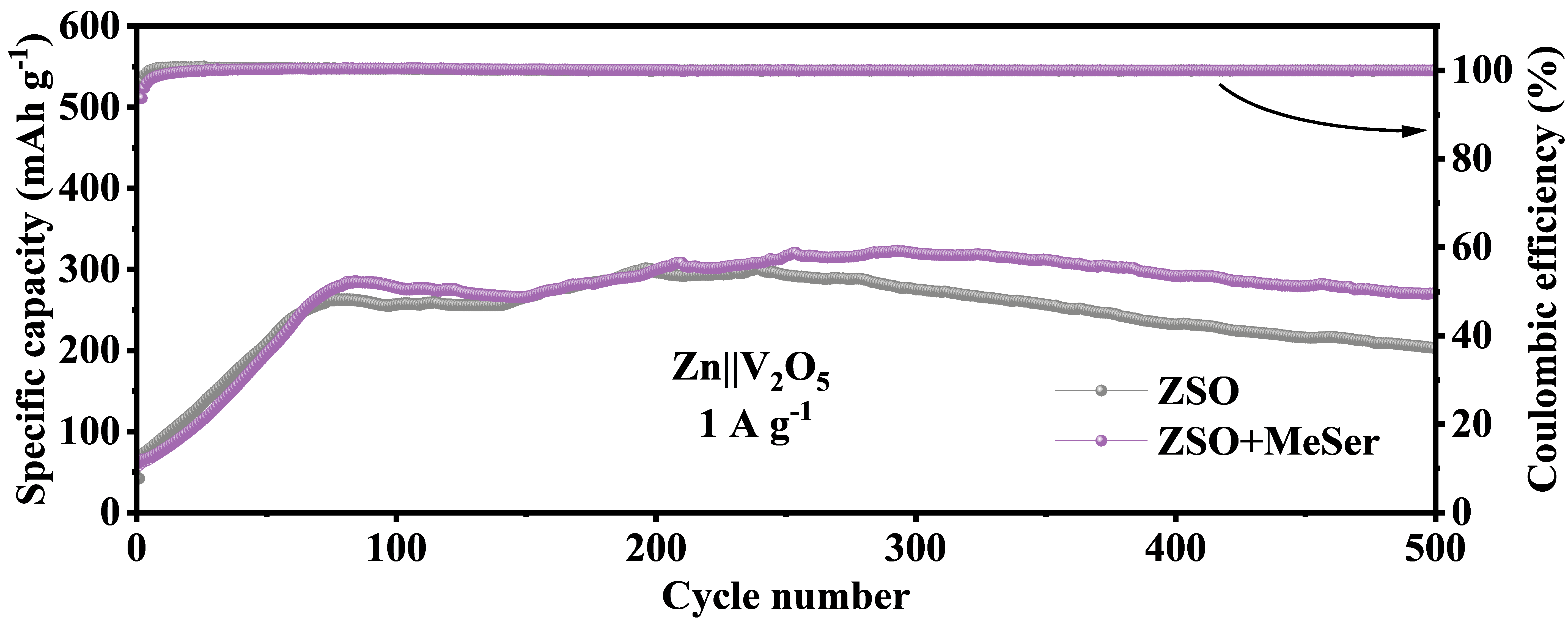


Figure S25. Long-term cycling performance of Zn||V_2_O_5_ full cells at 1 A g^−1^ in different electrolytes.

**References**

[1] G. Kresse, J. Furthmüller, *Computational materials science* **1996**, *6*, 15-50.

[2] J. P. Perdew, K. Burke, M. Ernzerhof, *Physical review letters* **1996**, *77*, 3865.

[3] S. Grimme, J. Antony, S. Ehrlich, H. Krieg, *The Journal of chemical physics* **2010**, *132*.
